# CASCADE, a platform for controlled gene amplification for high, tunable and selection-free gene expression in yeast

**DOI:** 10.1038/srep41431

**Published:** 2017-01-30

**Authors:** Tomas Strucko, Line Due Buron, Zofia Dorota Jarczynska, Christina Spuur Nødvig, Louise Mølgaard, Barbara Ann Halkier, Uffe Hasbro Mortensen

**Affiliations:** 1 Eukaryotic Molecular Cell Biology, Section for Eukaryotic Biotechnology, Department of Systems Biology, Technical University of Denmark, Søltofts Plads, Building 223, 2800 Kongens Lyngby, Denmark; 2 DynaMo Center, Department of Plant and Environmental Sciences, University of Copenhagen, Thorvaldsensvej 40, 1871 Frederiksberg C, Denmark

## Abstract

Over-expression of a gene by increasing its copy number is often desirable in the model yeast *Saccharomyces cerevisiae*. It may facilitate elucidation of enzyme functions, and in cell factory design it is used to increase production of proteins and metabolites. Current methods are typically exploiting expression from the multicopy 2 μ-derived plasmid or by targeting genes repeatedly into sequences like Ty or rDNA; in both cases, high gene expression levels are often reached. However, with 2 μ-based plasmid expression, the population of cells is very heterogeneous with respect to protein production; and for integration into repeated sequences it is difficult to determine the genetic setup of the resulting strains and to achieve specific gene doses. For both types of systems, the strains often suffer from genetic instability if proper selection pressure is not applied. Here we present a gene amplification system, CASCADE, which enables construction of strains with defined gene copy numbers. One or more genes can be amplified simultaneously and the resulting strains can be stably propagated on selection-free medium. As proof-of-concept, we have successfully used CASCADE to increase heterologous production of two fluorescent proteins, the enzyme β-galactosidase the fungal polyketide 6-methyl salicylic acid and the plant metabolite vanillin glucoside.

*Saccharomyces cerevisiae* is a widely used eukaryotic model organism and an increasingly popular cell factory for production of compounds ranging from small metabolites like ethanol to proteins like insulin^1^,^2^,^3^. To optimize yields and to tune metabolic pathways for a balanced flux it is often necessary to increase copy numbers of relevant genes for the process. In *S. cerevisiae*, multi-copy expression is most frequently achieved via use of self-replicating, 2 μ-based plasmids^4^. However, these plasmids fluctuate widely in copy number during propagation since they are not inherited as stably as chromosomes; this instability is a major cause of productivity loss in long-term cultivations^5^,^6^,^7^.

Genomic integrations are much more stable, but to meet plasmid-based expression levels, it is necessary to integrate multiple copies of each gene. This can be achieved by time-consuming repetitive rounds of genetic transformation into unique sites or by multiple insertions of the gene targeting substrate into repeated sequences, like rDNA, Ty, and δ-sequences, which are naturally occurring in the yeast genome^8^,^9^,^10^,^11^,^12^,^13^,^14^,^15^,^16^. Strains generated in the latter manner may still be unstable as the gene copies often insert in tandem repeat arrangements even when the targeted repeats are dispersed throughout the genome; hence, copies may be lost due to direct repeat recombination (DRR).

Boeke and co-workers have exploited the natural mechanism of transposon migration to successfully amplify genes. By inserting relevant genes into a Ty retrotransposon the gene was subsequently amplified by inducing transposition. In this manner strains with up to ten integrated gene copies were generated^17^. A drawback of this amplification technique is its dependence on highly error prone reverse transcriptase, and amplified gene copies are therefore likely to contain mutations. Moreover, the DNA fragment size that can be efficiently amplified by this method is likely low as the amplification rate and copy number decreased substantially when the size of cargo DNA was increased from 1 kb to 4.6 kb^16^.

Here we describe a gene amplification platform, CASCADE, which enables fast construction of stable strains with defined copy numbers of amplicons ranging from one to nine copies in the yeast *S. cerevisiae*. Inspired by homing endonucleases from mobile group I introns and mating-type switching in yeast^18^,^19^,^20^,^21^, gene amplification is achieved through double-strand break (DSB) stimulated gene conversion^22^ between gene acceptor cassettes (GA cassettes), which are present in defined numbers in individual gene acceptor starter strains (GAS strains), and a donor amplicon sequence that may contain one or more genes.

We demonstrate that amplicons containing one or more genes can be efficiently introduced into GAS strains and subsequently amplified to produce strains with up to nine integrated copies. Moreover, we show that our platform can be used to identify and remove bottlenecks in biosynthetic pathways to speed up cell factory optimization.

## Results

### Design and construction of the gene amplification system

**Figure 1:**
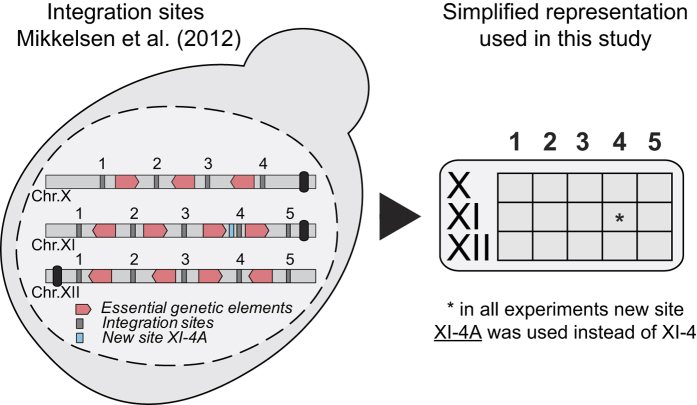
Genomic integration sites used for CASCADE platform. The integration sites have been previously selected and characterized by Mikkelsen^23^, with the exception of site XI-4A that replaces XI-4. The integration sites are organized in three clusters on chromosomes X, XI and XII. Moreover, individual integration sites are separated by essential genes. This feature is beneficial in cases where integrations sites contain identical genes or gene elements, e.g. promoters. Hence, if gene copies are lost due to DRR, such recombinants cannot propagate in the culture.

**Figure 2:**
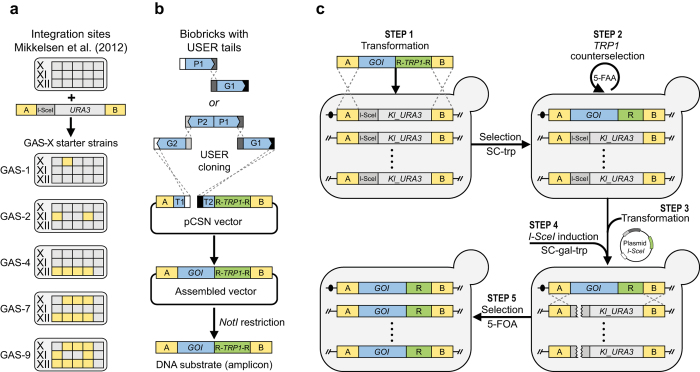
Overview of the CASCADE system. (**a**) The location of integrated GA cassettes in various GAS-X starter strains. (**b**) Assembly of the DNA “amplicon”. Promoters (P1, P2) and open reading frames (G1, G2) are assembled via USER cloning into the pCSN backbone, harboring terminators (T1, T2), selection marker *TRP1* and targeting regions A and B. The amplicon consisting of assembled gene/s of interest (GOI) is released from the plasmid by *NotI* restriction. (**c**) Five step gene amplification procedure (for details, see text).

The CASCADE platform for controlled gene amplification is based on defined expression loci in intergenic regions of the genome, which we have previously characterized for their ability to support high expression levels and accept foreign DNA without influencing fitness^23^. Integrations sites are clustered, and individual integration sites are separated by essential genes, which is a feature that prevents strains that have lost amplified genes through DRR from propagating in the culture. We have expanded the collection with two additional integrations sites to support the work presented below; one that clusters with the sites on chromosome XI, see Fig. 1 and Supplementary Figure 1, and one on chromosome VII, see Methods. GA cassettes, see Fig. 2a, containing an I-SceI cut site and a *Kl_URA3* marker flanked by recombination sequences ‘A’ and ‘B’ were introduced into selected subsets of these expression loci to produce GAS strains (GAS-X strains, where X indicates the number of GA cassettes in a given strain). Specifically, *MAT***a** and *MAT*α starter strains containing 1–5, 7, and 9 GA cassettes were constructed. The sequence to be amplified, the amplicon, is assembled in a specific gene-targeting vector, pCSN (this work), by USER cloning (Fig. 2b). As a result, the amplicon is inserted into a gene targeting substrate containing a *TRP1* selection marker, which is flanked by short repeats, and sequences A and B, which will target the entire substrate into one of the integrated GA cassettes by homologous recombination (HR). Increasing the genomic amplicon copy number is achieved in five simple steps, see Fig. 2c. In *step **1***, the gene targeting substrate is liberated from the pCSN vector backbone and used for transformation of one or more GAS-X strains (containing different numbers (X) of GA cassettes and/or different mating types) using the *TRP1* marker for selection. Importantly, the *Kl_URA3* marker gene in GA cassettes is lost at sites where the amplicon integrates. In *step **2***, the *TRP1* marker gene is recycled by selecting strains that have lost *TRP1* by DRR on medium containing 5-FAA. In *step **3***, a 2 μ-based plasmid containing a galactose inducible *I-SceI* gene is introduced into strains selected in *step **2*** using *TRP1* for selection. In *step **4***, by plating the strains on galactose medium, gene amplification is induced by I-SceI, which cuts all remaining GA cassettes. The A and B sequences of the broken GA cassettes mediate gene amplification, as intact amplicons (which are flanked by matching A and B sequences) are used as a template for repair. In *step **5***, strains in which all GA cassettes have been replaced by the amplicon are selected on medium containing 5-FOA, hence, exploiting that *Kl_URA3* genes embedded in GA cassettes are eliminated as amplicons transfer into each broken site by gene conversion. Since the 5-FOA medium contains tryptophan, most strains isolated are already cured for the I-SceI plasmid. In remaining cases, plasmid-free strains can easily be selected on medium containing 5-FAA.

### Using CASCADE for controlled amplification of *lacZ* and a *RFP*::*CFP* gene pair

**Figure 3:**
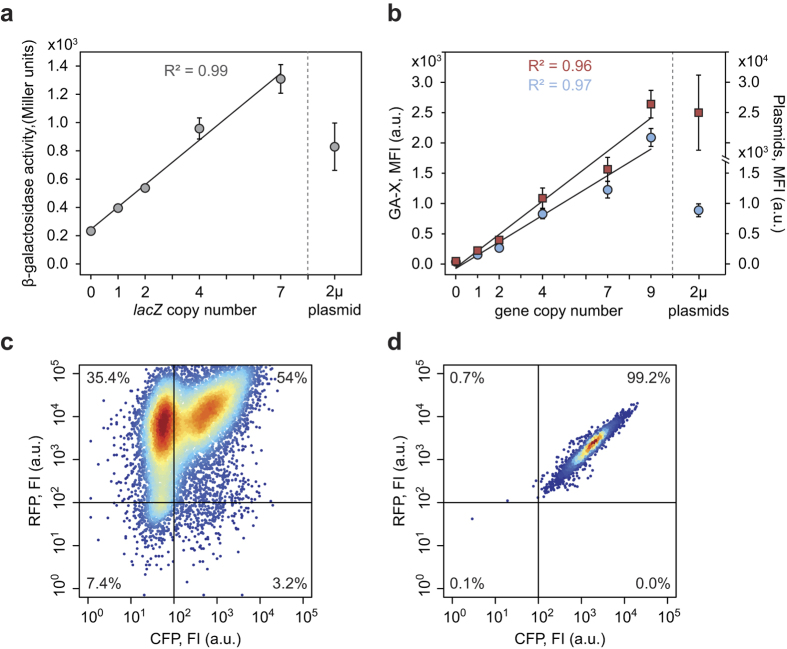
Impact of gene copy numbers on relative expression levels. (**a**) The activity of β-galactosidase as a function of the copy number of an integrated *lacZ* gene, and when expressed on a self-replicating 2 μ-derived plasmid. (**b**) Mean fluorescence intensities (MFI) of the red and cyan fluorescent proteins (RFP, red squares, and CFP, blue circles) as a function of the copy number of integrated *RFP* and *CFP* genes, and when expressed on self-replicating 2 μ-derived plasmids. (**c**) Density scatter plot of RFP signal as a function of the CFP signal when *RFP* and *CFP* were expressed on two different 2 μ-derived plasmids. (**d**) Density scatter plot of RFP signal as a function of the CFP signal when *RFP*::*CFP* was amplified to 9 copies in strain GA9-CR. Error bars ±SD, n = 3.

To test whether CASCADE can be used to amplify genes in a defined manner, we first constructed gene targeting vectors harboring amplicons containing either a *lacZ* gene controlled by an *ACT1* promoter or a C*FP*::*RFP* gene pair controlled by the bidirectional (*TPI1-ACT1*) promoter, see Methods. *lacZ* and *CFP*::*RFP* amplicons were introduced into GAS-X strains containing up to seven and nine GA cassettes resulting in four GAX-BG (X = 1, 2, 4 and 7) and in five GAX-CR (X = 1, 2, 4, 7 and 9) strains, respectively. After gene amplification steps ***1***–***5***, strains resistant to 5-FOA were easily obtained in all amplification experiments. For each strain, 4–10 colonies were typically picked and examined by PCR. In the vast majority of the cases, these analyses showed that amplification was complete. GAX-BG strains were analyzed for β-galactosidase activity, and we found that this activity increased linearly to *lacZ* copy numbers (Fig. 3a). Similarly, with GAX-CR strains the red and cyan mean fluorescent intensity (MFI) increased proportionally to *CFP*::*RFP* copy numbers (Fig. 3b).

We also compared the activity levels of GAX-BG and GAX-CR strains to strains where the *lacZ* gene was harbored on a single 2 μ-derived plasmid and the *CFP* and *RFP* genes on two different 2 μ-derived plasmids (Fig. 3a and b). The GA7-BG and GA9-CR strains produced significantly more β-galactosidase and CFP activity, respectively, as compared to the strains containing the corresponding *lacZ* and CFP 2 μ-derived plasmids (p < 0.05 and p < 0.005, respectively). Hence, CASCADE can be used to construct strains that are better producers than strains expressing the same two genes from 2 μ-derived plasmids. However, for production of RFP, the MFI obtained with the GA9-CR strain was approximately 10-fold lower than that obtained with the strain harboring the *RFP* 2 μ-derived plasmid. Hence, in this case, it will be difficult to generate a strain with a number of integrated *RFP* gene copies that is sufficiently high to beat RFP production based on this 2 μ-derived plasmid.

In metabolic engineering projects, it is often necessary to co-express a large number of genes to achieve the desired product. In many cases this has been achieved by transforming a host strain with more than one 2 μ-derived plasmid^4^. In this context, we have previously reported that individual cells within a population of a strain harboring 2 μ-derived plasmid contains very different protein levels^24^,^25^. Moreover, different 2 μ-derived plasmids appear to segregate largely independently, and thus, levels of a proteins encoded by these plasmids will result in populations where individual cells contain very different combinations of protein levels. Consequently, optimization of a cell factory based on multiple 2 μ-derived plasmids is highly complex as the enzymes supporting the newly implemented pathway will be unbalanced in the vast majority of the cells in the population. To investigate how well gene activities in a simple two-gene system, *CFP*::*RFP*, correlate in a population of cells containing multiple integrated copies of each gene, we measured cyan and red MFIs in individual cells in GAX-CR strains. Moreover, we compared these levels to those obtained with a strain that harbors the *CFP* and *RFP* genes on two different 2 μ-derived plasmids. In agreement with our previous results^24^, the strains expressing *CFP* and *RFP* from 2 μ-derived plasmids contained cells with cyan and red fluorescence signals varying from very high to no signal. In fact only 54% of the cells in the population fluoresced in both colors. Therefore, a cell containing high CFP signal levels may contain high, medium, or low of RFP signal levels, and *vice versa;* as result, the population consists of cells among which relative CFP and RFP levels vary dramatically (Fig. 3c). In contrast, in the GA9-CR strain >99% of the cells fluorescence in both colors, and CFP and RFP signal levels correlate well for individual cells; the vast majority of cells yield data points close to a straight line (R^2^ = 0.96) when the signal of RFP is plotted against that of CFP (Fig. 3d). Similar expression trends of both colors were observed in strains with lower copy numbers of the *CFP*::*RFP* (Supplementary Figure 2 and Supplementary Table 1). Furthermore, we assessed the production stability of CFP and RFP in strain GA9-CR and other CASCADE strains during long term cultivations and found that they retained initial fluorescence levels of the two reporter proteins after 40 generations in non-selective media. In contrast, with cells expressing *CFP* and *RFP* from 2 μ-derived plasmids, both CFP and RFP signals are reduced to less than one % of the initial fluorescence levels after 40 generations in non-selective medium (See Supplementary Note).

### Heterologous polyketide production by CASCADE

**Figure 4:**
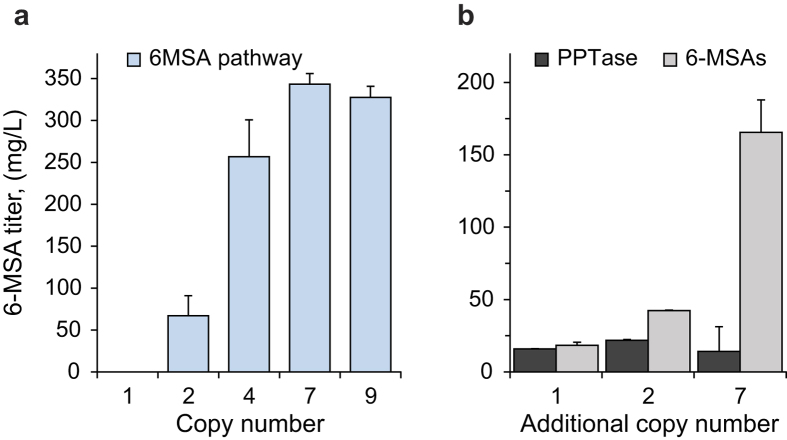
Final titers of 6-methyl salicylic acid (6-MSA) in the supernatant after 96 h of cultivation. (**a**) Production of 6-MSA as a function of the copy number of the 6-MSAS::PPTase gene pair. (**b**) Titration of the 6-MSA metabolic pathway using CASCADE. Filled bars – 6-MSA production in strains containing one 6-MSAS::PPTase gene pair plus additional copies of the PPTase gene; grey bars – 6-MSA production in strains containing one 6-MSAS::PPTase gene pair plus additional copies of the 6MSAS gene. Error bars ±SD, n = 3.

Next, we examined whether CASCADE can be used to optimize production of a specific metabolite using production of 6-methylsalicylic acid (6-MSA) as a model system. 6-MSA is a simple fungal polyketide, which has previously been successfully produced in *S. cerevisiae*^26^,^27^. Formation of 6-MSA is catalyzed by a single enzyme, the type I iterative polyketide synthase 6-MSAS, using acetyl-CoA and malonyl-CoA as substrates^28^. However, type I polyketide synthases are formed as apoenzymes each of which is converted into the active holoenzyme by addition of a phosphopantethein moiety by a phosphopantetheinyl transferase, PPTase^29^; as *S. cerevisiae* does not produce polyketides, yeast-based cell factories for 6-MSA production have been equipped with genes encoding 6-MSAS and a PPTase originating from filamentous fungi^26^,^27^. In our setup, we equipped 6-MSAS and PPTase genes from *Penicillium patulum* and *Aspergillus nidulans* with bi-directional promoter (*TEF1-PGK1*) and terminators (*CYC1-ADH1*), see Methods, forming an amplicon totaling 10.1 kbp. GAS-X strains (X = 1, 2, 4, 7 and 9) were transformed with this amplicon, which was then amplified as described above. The resulting strains, GAX-6MPP, were analyzed for production of 6-MSA by sampling supernatants after 16, 48, and 96 hours of batch cultivation. When the individual strains were compared, 6-MSA production increased dramatically at all time points as the number of amplicons were increased from one to seven, see Fig. 4a and Supplementary Figure 3. The additional two amplicons in the GA9-6MPP strain did not further increase 6-MSA yields, suggesting that 6-MSAS and/or PPTase levels do not limit 6-MSA production in the strain with seven copies.

We next applied CASCADE to determine whether increased 6-MSA production is due to increased 6-MSAS or PPTase levels. To this end, we exploited that we have constructed GAS-X strains in both mating types, setting the stage for a rapid combinatorial approach to investigate different gene copy number combinations in a setup based on diploid cells, see Methods and Supplementary Figure 4. Amplicons containing either the 6-MSAS or the PPTase gene were transformed and amplified to 1, 2 and 7 copies in GAS-X *MAT***a** strains and mated to a *MAT*α strain (XI2_6MPP) containing a single copy of the *6MSAS*::*PPT* gene pair. As a result, one set contains diploid strains with a single copy of the 6-MSAS gene and a total of either 2, 3, or 8 copies of the PPTase gene; and another set contains strains with one copy of the PPTase gene and a total of either 2, 3 or 8 copies of the 6-MSAS gene. The diploid strains were batch fermented, and 6-MSA levels determined after 96 hours. With all strains in the first set, 6-MSA levels were constant (Fig. 4b). In contrast, with strains in the second set, 6-MSA levels increased in a manner that positively depended on the number of 6-MSAS gene copies harbored by the strain. These results show that activation of the 6-MSAS apoenzyme by PPTase is not limiting 6-MSA production in these 6-MSA producing cell factories.

### Optimizing heterologous production of vanillin glucoside by CASCADE

**Figure 5:**
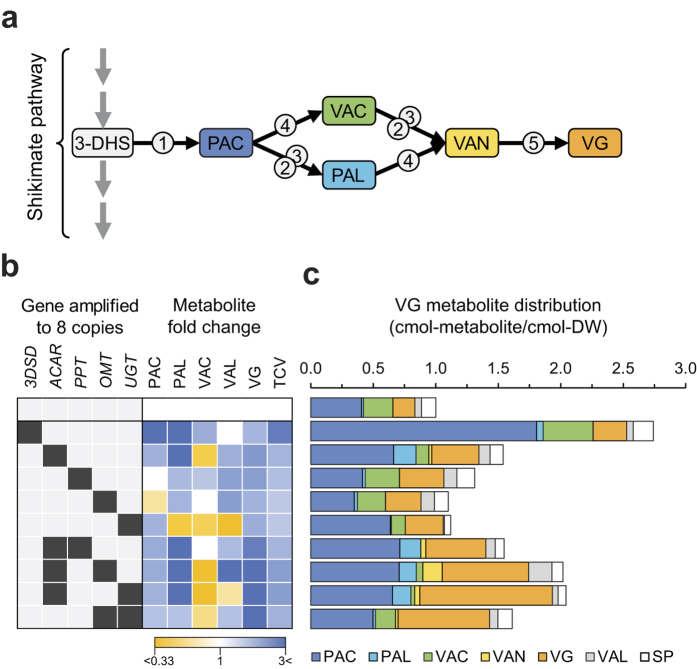
Vanillin glucoside (VG) production in diploid *S. cerevisiae* strains containing selected gene amplifications. (**a**) Schematic representation of the *de novo* biosynthetic pathway for VG production in *S. cerevisiae.* The black arrows depict reactions catalyzed by heterologous enzymes, and grey arrows indicate reactions catalyzed by yeast enzymes. Numbers in grey open circles represent specific enzymes; (1) 3-dehydroshikimate dehydratase (3DSD; from *Podospora anserina*), (2) aromatic carboxylic acid reductase (ACAR; *Nocardia iowensis*), (3) phosphopantetheine transferase (PPTase*; Escherichia coli*), (4) O-methyltransferase (OMT; *Homo sapiens*), and (5) UDP-glycosyltransferase (UGT; *Arabidopsis thaliana*. Colored boxes show metabolites of the pathway: 3-DHS – 3-dehydroshikimic acid; PAC – protocatechuic acid; PAL – protocatechuic aldehyde; VAC – vanillic acid; VAN – vanillin; VAL –vanillin alcohol (a shunt product generated from VAN by endogenous alcohol dehydrogenases); and TCV – total carbon ending in the VG pathway. (**b**) Heat map representing metabolic perturbations at the 60 h time points of the VG pathway with strains containing selected VG pathway gene amplifications (black boxes). The absence of VAN in the heat map is due to the lack of detectable VAN in the reference strain preventing the analysis. (**c**) The VG pathway metabolite distribution at 60 h normalized to the reference strain, which is a diploid strain containing one copy of each heterologous gene; n = 2. SP – VG pathway side products (sum of isovanillin and isovanillic acid).

We and others have previously established yeast-based cell factories for vanillin glucoside (VG) production^30^,^31^,^32^. The synthetic VG pathway is based on five heterologous genes, see Fig. 5a. Note that in the VG pathway, ACAR, like 6-MSAS, needs to be activated by a PPTase. Metabolite profiling of the VG cell factories showed production of several VG pathway related intermediates, some of which were made in larger quantities than VG. These results strongly suggest that the VG pathway is unbalanced with respect to the levels of individual enzymatic activities in the pathway. Moreover, the reported yields of VG and its related metabolites indicate that there is a potential to increase the flux from the shikimate pathway towards VG^30^,^31^,^32^,^33^. We therefore applied the CASCADE system in an attempt to identify and reduce bottlenecks to increase the carbon flux into the synthetic VG pathway to improve VG yields.

First, our gene amplification platform was used to create a set of five haploid strains each of which contains seven copies of one of the five VG pathway genes (see Methods and Fig. 5a). The mating types of these strains were opposite to that of the VG cell factory we have previously constructed; and five diploid strains, where each strain contains one copy of all genes in the VG pathway as well as additional seven copies of one of the other five genes in the VG pathway, were made by simple mating (see Methods and Supplementary Figure 4). The haploid VG cell factory was also mated to a strain containing no additional VG genes to form a sixth diploid strain, which served as a reference strain. In agreement with previous results, metabolite profiling of the supernatant obtained with the diploid reference strain after fermentation in shake flasks demonstrated that within the pool of VG related metabolites measured, the intermediates PAC, VAC, and VG constituted 41%, 24%, and 18%, respectively (Fig. 5b). Hence, as with haploid strains, the VG pathway is also unbalanced in the diploid cell factory, setting the stage for optimization of VG production through specific gene amplification. We therefore analyzed the set of diploid strains for formation of VG metabolites.

The highest overall change, 2.7-fold, in the amount of total carbon entering the VG pathway was measured in the strain overexpressing the gene encoding 3DSD, which catalyzes the first step in in the VG pathway, Fig. 5b and c, and Supplementary Table 2. The increase in carbon flux into the VG pathway was mostly due to a 4.5-fold increase in the first intermediate, PAC, in the VG pathway. Smaller increases in VAC (1.7-fold) and in VG (1.5-fold) production were also observed. A noticeable increase in the VG pathway flux was also observed for strains with multicopy expression of the gene encoding ACAR and for the strain with multicopy expression of the PPTase, which is responsible for the activation of ACAR. In these strains, the total increase is due to elevated levels of PAC (1.6-fold) and VG (2.1-fold), respectively. Importantly, the final yield of VG was higher with all diploid strains with multicopy expression of a single gene in the VG pathway as compared to the diploid reference strain.

The fact that individual multicopy expression of genes in the VG pathway leads to higher VG and VG related metabolite production indicates that increased flux into the VG pathway can be achieved by capturing more carbon from the shikimate pathway as well as by creating metabolic sinks in the VG pathway by increasing the concentrations of enzymes acting early or late in the VG pathway, respectively. We therefore investigated whether VG production could be further stimulated by simultaneous multicopy expression of two of the genes in the VG pathway. For this purpose we adopted the same strategy as for constructing the diploid cell factories described above except that amplicons containing two VG genes were simultaneously amplified in our platform strains. First we investigated the impact of the gene pair encoding ACAR and PPTase on VG production. In this case, multiple copies of both genes yielded a metabolite profile essentially identical to that achieved with multicopy expression of only the gene encoding ACAR, indicating that PPTase is not a limiting enzyme in these strains. Consequently, we next focused on combining multiple copies of the ACAR gene with multiple copies of genes encoding enzymes acting later (or in the case of the gene encoding OMT, potentially earlier) in the VG pathway; two such strains were constructed together with a third diploid strain that had multiple copies of OMT and UGT. All three strains produced more VG than any of the strains that individually express a single VG pathway gene in multiple copies. The largest effect was observed with the strain with multiple copies of the gene pair encoding ACAR and UGT, and in this case, a synergistic increase in VG production was achieved. In fact, 6-fold more VG was produced as compared to the diploid reference strain. Moreover, the VG metabolite profile appears much more balanced in this diploid strain, as VG constitutes the major product, and as the percentage of VG in the total pool of measured metabolites derived from the VG pathway is increased from 18% to 52%.

## Discussion

We have successfully demonstrated that CASCADE can be used to amplify chromosomal gene copy numbers in a robust and controlled manner by taking advantage of efficient DNA DSB repair by HR in *S. cerevisiae*. Hence, we have demonstrated that CASCADE can be used to increase intracellular protein levels, to identify metabolic pathway bottlenecks, and to optimize production of metabolites resulting from a multi-gene pathway.

Current methods aiming at increasing gene copy number are typically based on 2 μ-derived plasmids or on targeting genes into repeated sequences like Ty or rDNA^8^,^9^,^10^,^11^,^12^,^13^,^14^,^15^,^16^. In both cases, high gene expression levels are often reached, and production levels usually drop over time unless selection is applied to maintain copy numbers^8^,^9^,^10^,^11^,^12^,^13^,^14^,^15^,^16^. Moreover, the resulting strains are genetically ill-defined and they cannot in a straight forward manner be used to titrate and identify the optimal gene dose(s) required to balance and optimize metabolic pathways.

In contrast, CASCADE offers strains containing defined gene copy numbers integrated at defined chromosomal sites. Importantly, strains developed by CASCADE are likely to be genetically stable and no selection is required to maintain copy numbers. Several precautions are made to minimize gene loss and other genetic rearrangements that may reduce productivity. Specifically, amplicon loss by DRR is accompanied by cell death, so cells with a lower amplicon copy number will not propagate in the culture. We note that amplicon amplification may also happen as the result of DRR. However, as increased gene copy number most often leads to increased metabolic stress, such recombinants will grow slower than the original cells and are therefore unlikely to take over the population. Crossing over may also happen between amplicons on different chromosomes in GAS-7 and GAS-9 strains. However, the positions of the GA cassettes are designed to reduce viability of such recombinants. For example, in the case of GAS-7 strains, which contain GA cassette clusters on two chromosomes, inter-chromosomal crossing over will result in formation of a di-centric chromosome, which is lethal. As a result of these precautions, production of CFP and RFP remained unchanged for 40 generations.

So far we have amplified amplicons of sizes up to 10.1 kb (Supplementary Table 3) and demonstrated that it is possible to amplify two genes at the same time. We therefore envision that it will be possible to amplify and optimize even multi-gene pathways. If true, the challenge may be to insert a large number of genes into a GAS-X strain. However, it was recently shown that large pathways containing many different DNA fragments can be efficiently assembled by HR and integrated into a defined site, also by HR, in an event that is stimulated by an I-SceI or Cas9 induced DNA DSB at the locus^34^,^35^; and we believe that this method can easily be used in combination with CASCADE to increase production of complex metabolites.

Many different *S. cerevisiae* strain backgrounds are used in academic and industrial environments. Hence, it will be useful to implement CASCADE in other strain backgrounds than CEN.PK. Towards this goal, the bottleneck is the construction time required to build a set of GAS-X strains. However, this limitation can be potentially alleviated using multiplex CRISPR/Cas9 technology to reduce the numbers of transformation steps required for GAS strain construction^36^,^37^,^38^. Moreover Cas9 could be used to facilitate construction of GAS strains with more than nine GA cassettes. Since this strategy can also be used in many other organisms, we envision that CASCADE can be advantageously implemented in a broad range of prokaryotes and eukaryotes and used for cell factory construction, for research where different well-defined levels of expression of a gene is part of the research strategy, or in organisms where no multi-copy plasmid is available.

## Methods

### Cultivation media

To propagate *Escherichia coli* DH5α strains harboring the cloned plasmids, lysogeny broth (LB)^39^ supplemented with 100 mg/L of ampicillin (Sigma) was used.

For *S. cerevisiae* strains three types of medium was used. For genetic manipulations of yeast the synthetic complete (SC) medium was prepared as previously described by Sherman *et al*.^40^, with minor modifications (leucine concentration increased to 60 mg/L). All yeast transformants were selected on SC media missing the appropriate amino acid (denoted as SC-xxx, *e.g.* SC-trp if tryptophan is missing). For selection of the *TRP1* marker loss, yeast strains were grown on SC-5-FAA medium supplemented with 500 mg/L of 5-fluoroanthranilic acid (Sigma). Strains that lost the *URA3* marker were selected on SC-5-FOA medium supplemented with 30 mg/L uracil and 740 mg/L 5-fluoroorotic acid (Sigma). For induction of *pGAL*::*I-SceI* plasmids SC medium was modified by using 2% galactose instead of glucose as a carbon source (SCgal).

Yeast extract Peptone Dextrose (YPD) medium was used for routine cultivations and storage of the constructed yeast strains. The medium composition is as follows: 10 g/L of yeast extract, 20 g/L of peptone and 20 g/L of glucose. If medium had to be solidified 20 g/L of agar was added prior autoclavation.

For vanillin glucoside (VG) pathway titration experiments a defined minimal medium (MM) previously described by Verduyn *et al*.^41^ with 20 g/L glucose as a carbon source was used. The medium composition is as follows: 7.5 g/L (NH_4_)_2_SO_4_, 3 g/L KH_2_PO_4_, 0.75 g/L Mg_2_SO_4_, 1.5 mL/L trace metal solution, 1.5 mL/L vitamins solution, 0.05 mL/L Antifoam 204 (Sigma-Aldrich). The trace metal solution contains 3 g/L FeSO_4_·7H_2_O, 4.5 g/L ZnSO_4_·7H_2_O, 4.5 g/L CaCl_2_·6H_2_O, 0.84 g/L MnCl_2_·2H2O, 0.3 g/L CoCl_2_·6H_2_O, 0.3 g/L CuSO_4_·5H_2_O, 0.4 g/L NaMoO_4_·2H_2_O, 1 g/L H_3_BO_3_, 0.1 g/L KI and 15 g/L Na_2_EDTA∙2H_2_O. The vitamin solution includes 50 mg/L d-biotin, 200 mg/L para-amino benzoic acid, 1.0 g/L nicotinic acid, 1.0 g/L Ca-pantothenate, 1.0 g/L pyridoxine HCL, 1.0 g/L thiamine HCl and 25 mg/L minositol. To maintain a constant pH of around 5.6 the medium was buffered by adding 10 g/L succinic acid and 6 g/L NaOH. The 10X concentrated glucose solution was autoclaved separately, the 1000X concentrated vitamin solution was sterile filtered (pore size 0.2 μm Ministart^®^-Plus, Sartorius AG, Germany) and both added after autoclavation.

### Molecular cloning procedures

All plasmids, unless otherwise stated, were assembled using the uracil-specific excision reagent (USER™) following well established protocols^42^. DNA fragments that constituted promoters, genes, terminators and other DNA sequences were amplified by PCR using PfuX7^43^ polymerase with specific primers (see Supplementary Table 4). In cases where two DNA fragments had to be merged prior the USER cloning, PCR fusion was applied^44^. All plasmids used and constructed in this work are listed in the Supplementary Table 5. A schematic representation of plasmids construction is depicted in Supplementary Figure 5.

UP and DOWN targeting regions of the new XI-4A site were amplified with primers P1, P2 and P3, P4, respectively, and USER cloned to generate the pXI-4A plasmid in a manner described by Mikkelsen *et al*.^23^. The gene expression levels of the new integration sites were validated (see Supplementary Figure 1).

A set of plasmids that was used to build the GAS-X strains were constructed as follows. nine plasmid backbones each containing targeting sequences to a specific locus on chromosome X, XI or XII were amplified from relevant templates (pX-2, pX-3, pX-4, pXI-1, pXI-4A, pXII-1, pXII-2, pXII-3 and pXII-4) using primers quipped with standardized uracil containing tails. Each of the resulting sequences were independently fused with three PCR fragments (*fragment 1*: targeting sequence ‘A’ fused with I-SceI recognition site and 2/3 of *5*′*-URA3* marker, *fragment 2: TRP1* selection marker, *fragment 3*: 2/3 of *3*′*-URA3* marker fused with ‘B’ sequence) that constituted an AUTUB cassette. The targeting sequence ‘A’ (0.5 kb) was PCR amplified from genomic DNA (gDNA) of *Neurospora crassa*. Whereas, the targeting sequence ‘B’ (1 kb) was a result of PCR fusion of the two 500 base pair fragments where the first half was amplified from gDNA of *A. nidulans* and the second half from gDNA of *N. crassa*.

For integration of the GA cassette located on the chromosome VII a bi-partite linear fragment was constructed as follows. The UP and DOWN targeting sequences were amplified with P32, P33 and P34, P35 primers, respectively. The UP fragment then was fused to the PCR fragment harboring A::Isce-I::5′URA3, and the DOWN sequence to the 3′URA3::B fragment.

The pCSN plasmid that served as a basic vector for the assembly of DNA targeting substrates compatible with GAS strains was cloned in two steps. First, two PCR fragments (*fragment 1*: sequence ‘A’ fused with an *ADH1* terminator, *fragment 2*: ‘B’ sequence) were cloned into linearized p1_USER vector forming an intermediate p1_ATB plasmid. Importantly, tails fusing fragments 1 and 2 were designed in a way that would restore USER cloning cassette (AsiSI/Nb.BsmI). In the second round of cloning, terminator of the *TPR1* marker (*tTRP1*), was fused upstream of the *TRP1* marker sequence and cloned into linearized p1_ATB. In this case, the *tTPR1* is meant to serve as the direct repeat ‘R’ sequence for the counter-selection of the *TRP1* marker.

All pCSN based plasmids harboring gene(s) of interest (GOI) for the proof-of-concept and metabolic pathway titration experiments were constructed in a similar fashion. In all cases, one or two genes were cloned with uni- or bi-directional promoters into (AsiSI/Nb.BsmI) linearized pCSN vector using standardized cloning procedure described in ref. 23. The GOI were PCR amplified from appropriate plasmid templates using primers with specific uracil containing tails. The p*TEF1* and p*PGK1* promoters were amplified from pSP-G2 plasmid and the p*ACT1* and p*TPI1* promoters from gDNA of *S. cerevisiae* (CEN.PK background).

The 2 μ based multi-copy plasmids were constructed as follows. A fragment encoding *lacZ* under the control of the p*ACT1* promoter was made by PCR using pWJ1042-AZC as template. The PCR fragment was inserted into a *Bam*HI-*Eag*I linearized vector fragment of pESC-HIS to yield the plasmid pESC-HIS-BG. Similarly, 2 μ vectors expressing RFP and CFP were constructed by inserting a PCR fragment into a *Eag*I-*Sal*I linearized vector fragment of pESC-HIS or a *Bam*HI-*Eag*I vector fragment of pESC-URA to yield the plasmids pESC-HIS-RFP and pESC-URA-CFP, respectively. CFP was amplified from the plasmid pWJ1163, whereas RFP was amplified from the plasmid pWJ1350.

All constructed plasmids were validated by DNA sequencing (StarSEQ^®^ GmbH, Germany).

### Yeast strain construction

All *S. cerevisiae* strains used in this study are based on CEN.PK^45^ background and are listed in Supplementary Table 6. All genetic transformations of yeast were done using standard procedures described in ref. 46. Yeast mating, diploid sporulation and tetrad dissection was done following well established procedures described in ref. 47.

### CASCADE platform strains

The yeast strain collection establishing our CASCADE platform was generated as follows. Gene accepting starter (GAS) strains containing up to four integrated gene acceptor (GA) cassettes were constructed using standard genetic transformation. In the first round of transformation, *MAT***a** or *MAT*α strain was transformed with one out of nine linear AUTUB fragments flanked by up- and downstream sequences targeting to a defined location on chromosome X, XI or XII. The resulting transformants with an integrated AUTUB cassette were selected on SC-trp medium. Next, the strains that have lost *TRP1* marker from the AUTUB fragment by direct repeat recombination and, therefore, containing functional GA cassette were selected on SC-5-FAA medium. Once, the first GA cassette was integrated the transformation cycle was repeated using different AUTUB cassette until required number of the GA cassettes was achieved. As a result, a collection of GAS-1, -2, -3 and -4 strain variants with GA cassettes integrated on chromosomes X, XI and XII was generated (see Supplementary Figure 6 and Supplementary Table 6).

The GAS strains with more than four GA cassettes were built by mating, diploid sporulation and subsequent tetrad dissection. The GAS-7 strain variants were constructed by crossing GAS-4 (four GA cassettes on chromosome XII) with GAS-3 (three GA cassettes on chromosome X). Similarly, GAS-9 strains were generated by mating GAS-7 with GAS-2. Most importantly, each of the GAS-X strains were made in two mating types, one with *MAT***a** and the other *MAT*α (see Supplementary Figure 6 and Supplementary Table 6).

### General method for gene amplification by CASCADE

Schematic representation of gene amplification protocol is depicted in Supplementary Figure 7 where genomic amplification is achieved in the steps described below:

***STEP 1***. The GAS-X strain with a defined number of GA cassettes is transformed with the linear DNA targeting substrate (0.2–0.5 μg/μL) containing amplicon (assembled in “pCSN-GOI” plasmid). Transformed cells are plated on SC-trp medium and incubated at 30 °C for 2–3 days. Routinely, 4 single colonies from transformation plate are streak-purified on the selective medium for the following procedures.

***STEP 2***. The strains that have lost *TRP1* marker by direct repeat recombination are selected by streaking transformants (**step 1**) on plates with SC-5-FAA medium and incubating at 30 °C for approx. 3 days. Single colonies are then streaked on YPD and SC-trp (negative control) plates. Only colonies that grew on YPD and didn’t grow on SC-trp are considered for the next step.

***STEP 3***. Single colony from **step2** is transformed with the 2 μ-based plasmid pWJ1320-TRP (up to 0.5 μg/μL), which contains a galactose inducible *I-SceI* gene. The resulting transformants are incubated at 30 °C for 2–4 days on SC-trp medium. In this step it is important to adjust number of transformed yeast cells or concentration of pWJ1320-TRP to achieve approx. 50 to 200 single colonies per selection plate!

***STEP 4***. The gene amplification is induced by replica plating transformation plate from **step3** onto induction media SCgal-trp plates. The plates are incubated at 30 °C for 3–4 days or until yeast colonies appear.

***STEP 5***. To identify the colonies where all GA cassettes were replaced by the amplicon, the SCgal-trp plate from **step 4** is replica plated onto SC-5-FOA and incubated for 2–3 days at 30 °C. The resulting SC-5FOA plate is then replica plated onto YPD and SC-ura plates and incubated overnight at 30 °C. Colonies growing on YPD plates but not on SC-ura are plausible candidates with successfully amplified genes of interest. Routinely, 4 colonies are streak-purified on plates with SC-5-FOA medium and validated by colony PCR with the primer pairs specific to the amplification sites (see Supplementary Table 4). For example, to confirm the amplification in the site “X2” colony PCR was run with generic primer P116 (that anneals to the *ADH1* terminator region of an amplicon) and site specific primer P97(X2-F) that would result in an approx. 800 bp DNA fragment. No PCR fragment is obtained with the P116 and P97 primers if GA cassette in the site “X2” was not exchanged by an amplicon. In addition, to test if full length of amplicon was successfully integrated primers P97(X2-F) and P106(X2-R) were used, the size of PCR product varies according to the size of an amplicon.

### The proof of concept strains (*lacZ, CFP* and *RFP* expressing strains)

For the proof of concept experiments, the haploid strains with different copy numbers of integrated *lacZ* or *CFP*::*RFP* genes were constructed using the CASCADE method described above. More specifically, the β-galactosidase producing strains with up to seven copies of *lacZ* were constructed by transforming GAS-2E, GAS-4D and GAS-7A strains with linear fragment obtained by *NotI* restriction of pCSN-BG plasmid. After gene amplification and its validation the GA1-BG, GA2-BG, GA4-BG and GA7-BG strains were obtained. The 2M-BG strain expressing *lacZ* on a multi copy plasmid was generated by transforming the CEN.PK113-1C with pESC-HIS-BG and selecting on SC-his medium. Wild type CEN.PK113-1C strain was used as a negative control in β-galactosidase assays.

The strains producing cyan and red fluorescent proteins with up to 9 copies of integrated *CFP*::*RFP* gene pair were generated by transforming GAS-2D, GAS-4D, GAS-7F and GAS-9A strains with *NotI* linearized fragment from pCSN-CR plasmid. This resulted in strains denoted as GA1-CR, GA2-CR, GA4-CR, GA7-CR and GA9-CR. The strain 2M-CR was generated by co-transforming CEN.PK113-1C with the pESC-URA-CFP and pESC-HIS-RFP plasmids and subsequently selecting the cells on SC-ura-his medium.

### 6-MSA producing strains

The strains used in 6-MSA pathway titration experiments were constructed as follows. To generate the basic strain expressing *6-MSAS*::*PPT* gene pair the CEN.PK113-17A was transformed with *NotI* linearized pXI2-6MPP plasmid and selected on SC-ura. Thereafter, the *URA3* marker was counter selected by plating on SC-5-FOA medium resulting in strain XI2_6MPP. Strains expressing more than one copy of *6-MSAS*::*PPT* gene pair were generated by transforming GAS-1D, GAS-2D, GAS-4D, GAS-7F and GAS-9A with linearized fragment from pCSN-6MPP plasmid obtained by *NotI* digestion. After standard CASCADE amplification procedure the strains GA1-6MPP, GA2-6MPP, GA4-6MPP, GA7-6MPP and GA9-6MPP were obtained. Constructs with one, two and seven copies of *6-MSAS* or *PPT* were made by transforming GAS-1D, GAS-2D and GAS-7F with linear fragment from pCSN-6M or pCSN-PP plasmids, respectively. The resulting strains after CASCADE amplification were named GA1-6M, GA2-6M and GA7-6M, and GA1-PP, GA2-PP and GA7-PP, respectively.

Heterozygous diploid strains were generated using the scheme depicted in Supplementary Figure 4. The strains with various combinations of *6-MSA* and *PPT* gene copies were constructed by mating the XI2_6MPP with each of GAX-6M or GAX-PP (see Supplementary Table 7). A control diploid (without 6-MSA pathway) was constructed by mating CEN.PK113-11C and CEN.PK113-17A strains. All diploids were selected on SC-his-leu medium.

### Vanillin glucoside producing strains

Haploid strain XII_VG containing the full VG biosynthetic pathway was obtained by cross of CEN.PK110-16D and C-VG-aux^32^ strains, followed by sporulation and tetrad dissection. For the titration experiments, GAS-7F strain had to be modified by double deletion of *adh6* and *bgl1* genes in order to be compatible with the *de novo* VG pathway. This was done by crossing GAS-7F with TS086^32^ and isolating the GAS-7VG strain that was used as a starter strain for amplification of VG pathway genes. All haploid strains with seven copies of integrated GOI were constructed as follows; the GAS-7VG strain was individually transformed with different linear fragments obtained by digesting pCSN-VG## plasmids with *Not*I endonuclease. After amplification by CASCADE method nine strains (GA7-VG01, GA7-VG20, GA7-VG03, GA7-VG04, GA7-VG50, GA7-VG23, GA7-VG24, GA7-VG25 and GA7-VG54) were generated.

To analyze the effect of increased dosage of the VG pathway genes, diploid strains were constructed in a similar fashion as for 6-MSA experiments. In brief, the XII_VG strain was crossed with each version of the GA7-VG## strain and resultant diploids were selected on SC-his-ura plates. All diploids used in VG titration experiment are listed in Supplementary Table 7.

### Determination of β-galactosidase activity

Three single colonies of each strain expressing *lacZ* gene were inoculated into 5 mL of SC medium (SC-his for strain with a plasmid) and incubated O/N at 30 °C with 150 rpm agitation. Next day, 1 mL of the O/N cultures were inoculated into 9 mL of fresh SC medium and grown until mid-exponential phase. Thereafter, 5 mL samples were taken and β-galactosidase activity was determined according to standard methods^48^ with modifications described in ref. 23. Optical density of the assay samples were recorded using a Synergy2 multi-mode microplate reader (BioTek). The β-galactosidase activity was normalized to the total protein concentration determined by standard Bradford assay^49^.

### Flow cytometry analysis

Three single colonies of each strains expressing *CFP* and/or *RFP* genes were inoculated into 3 mL of SC medium (SC-his-ura for strains with plasmids) and grown O/N with 150 rpm shaking at 30 °C. 50 μL of each culture was transferred to 50 mL falcon tube containing 15 mL of fresh medium and incubated under the same conditions. When cultures had reached mid-exponential phase yeast cells were harvested and fixed with paraformaldehyde according to the following protocol. 2 mL of each culture was cooled on ice and subsequently centrifuged at 4 °C, 3000 × g for 2 min. Supernatant was removed and pellet was re-suspended in 200 μL of 2% paraformaldehyde. The mix was incubated on ice for 1 hour and subsequently centrifuged at 4 °C, 3000 × g for 2 min. Finally, the paraformaldehyde was removed and pellet was re-suspended in 200 μL PBS buffer. The fixed cells were stored at 4 °C until analysis by flow cytometry.

The samples were analyzed on a BD FACSAria II equipped with three solid state diode lasers: air-cooled Coherent^TM^ Sapphire^TM^ solid-state diode laser (488 nm, 100 mW), air-cooled Coherent^TM^ Yellow Green laser (561 nm, 100 mW), and an air-cooled Coherent^TM^ Deep Blue laser (445 nm, 50 mW). The following filters were used: mCFP-A and PE-Cy5-A for analysis of emission from CFP and RFP, respectively. Signal compensation was performed according to manufacturer’s protocol (BD FACSAria II User’s manual). For non-fluorescent (negative control) wild-type CEN.PK113-1C strain was used. Fluorescence threshold for both CFP and RFP signals was set to 100 fluorescence arbitrary units. For each sample 10000 cells (events) were recorded at an approximate rate of 2000 counts/sec. Generated data sets were analyzed and interpreted by open source software (Flowing Software v2.5.1) developed by Perttu Terho. Heat scatter plots were produced using open source R package *LSD* (https://cran.r-project.org/web/packages/LSD).

### Cultivation of 6-MSA producing strains

Four single colonies of each strain expressing 6-MSA pathway or its single genes were inoculated in 3 mL of YPD medium and grown O/N with 150 rpm shaking at 30 °C. An appropriate volume of each O/N culture was centrifuged at 4000 g for 5 min and pellets resuspended in fresh YPD medium. The resuspended cultures were diluted to a starting OD_600_ = 0.01 in 5 mL of YPD. Thereafter, 750 μL aliquots of each diluted culture were transferred into wells of white Krystal 24-well clear bottom microtiter plates (Porvair Sciences, Leatherhead, UK). Plates were incubated in Growth Profiler 1152 (Enzyscreen, Haarlem, The Netherlands) at 30 °C with constant shaking at 225 rpm. Samples for 6-MSA quantification and OD estimation were taken at three time points 16, 48 and 96 hours.

### Cultivation of vanillin glucoside producing strains

Pre-inoculum of the diploid strains for the VG titration were incubated O/N in 3 mL of SC-his-ura medium with constant agitation of 150 rpm at 30 °C. The shake-flask cultivations were performed in 500 mL flasks containing 50 mL of buffered MM medium (pH = 5.6), which were incubated at 30 °C on an orbital shaker set to 150 rpm. The flasks were inoculated with an initial OD_600_ = 0.01 and sampled after 60 h the end of cultivation. Two biological replicates were used for each VG producing strain.

The biomass concentration was determined by cell dry-weight (DW) measurements as described in ref. 50 using polyethersulfone (PES) filters with a pore size of 0.45 μm Montamil^®^ (Membrane Solutions, LLC). The filters were pre-dried in a microwave oven at 150 W and weighed. 5 mL of cultivation broth was filtered and then washed with three volumes of distilled water. Thereafter, the filters with biomass were dried in the microwave oven at 150 W for 20 min and cooled down in a desiccator for a minimum of 2 hours. Finally, the filters with biomass were weighed and the cell DW was determined. For the conversion of biomass to a C-mol units the molecular weight for biomass of 26.4 g/C-mol was considered^51^.

### HPLC methods for extracellular metabolite analysis

Samples for the quantification of vanillin glucoside (VG) and its pathway catabolites were prepared as follows: 500 μL of fermentation broth and 500 μL of 96% EtOH were carefully mixed by vortexing and centrifuged at 12000 × g for 2 min, the supernatant was transferred to a new tube and stored at −20 °C until further analysis. Extracellular vanillin β-D-glucoside (VG), vanillin (VAN), protocatechuic acid (PAC), protocatechuic aldehyde (PAL), vanillic acid (VAC), isovanillin (IVAN), isovanillic acid (IVAC) and vanillin alcohol (VAL) were quantified using Agilent 1100 series equipment with a Synergi Polar-RP 150*2 mm 4 u column (Phenomenex). A gradient of acetonitrile (ACN) with 1% tetra-fluoroacetic acid (TFA) and water with 1% TFA at a constant flow rate of 0.5 mL/min was used as mobile phase. The elution profile was as follows: 5% ACN for 1 min, 5% ACN to 30% ACN for 8 min, 30% ACN to 100% ACN for 1 min, 100% ACN for 1 minute, 100% ACN to 5% ACN for 3 min. The column was kept at 40 °C and metabolite detection was performed using a UV diode-array detector set to 230 and 280 nm.

Samples for the quantification of 6-methylsalicylic acid (6-MSA) were prepared as follows: 500 μL of fermentation broth was centrifuged at 4000 × g for 5 min, the supernatant was transferred to a 1.5 mL Eppendorf tube and stored at −20 °C until further analysis. The supernatant was analyzed using HPLC method developed by (Knudsen *et al*., unpublished results). Briefly, the 6-MSA was quantified using a Dionex Ultimate 3000 UHPLC coupled with an ultimate 3000 RS diode array detector (Dionex, Germering, Germany) equipped with a Poroshell 120 phenyl hexyl 2.1 × 100 mm, 2.7 μm (Agilent, Santa Clara, CA/USA) column by gradient elution at 60 °C and a flow rate of 0.8 mL/min starting at 15% acetonitrile and MiliQ water and ramped up to 100% in 7 min. before returned to the starting point in an additional 5 min. Quantification was performed using an eight-level external calibration curve with concentrations from 1–300 mg/L at a wavelength of 210 nm.

### Genetic stability assay

Triplicates of 2M-CR and GA-9CR strains expressing *CFP* and *RFP* genes were inoculated to 500 mL shake flasks containing 100 mL of SC (for plasmid strains also SC-ura-his) medium and incubated at 30 °C with 150 rpm agitation. After 24 hours of cultivation an appropriate volume of culture from each flask was transferred to a new flask with fresh medium to a starting OD_600_ ~ 0.1. The cycle was repeated for five consecutive days and 2 mL samples for flow cytometry analysis were collected once a day during mid-exponential growth phase. Samples were processed using paraformaldehyde fixation method described above. All accumulated samples were analyzed in flow cytometer BD FACSAria II at the same day using the settings described above.

## Additional Information

**How to cite this article**: Strucko, T. *et al*. CASCADE, a platform for controlled gene amplification for high, tunable and selection-free gene expression in yeast. *Sci. Rep.* **7**, 41431; doi: 10.1038/srep41431 (2017).

**Publisher's note:** Springer Nature remains neutral with regard to jurisdictional claims in published maps and institutional affiliations.

## Supplementary Material

Supplementary Information
