# Supplementary material for: CASCADE, a platform for controlled gene amplification for high, tunable and selection-free gene expression in yeast
Source: Sci Rep. 2017 Jan 30;7:41431. doi: 10.1038/srep41431 (PMC5278378; doi:10.1038/srep41431)
Supplement: Supplementary Information [file srep41431-s1.pdf]

CASCADE, a platform for controlled gene amplification for high, tunable and selection-free gene expression in yeast

Tomas Strucko[1,¥], Line Due Buron[1,¥], Zofia Dorota Jarczynska[1], Christina Spuur Nødvig[1], Louise Mølgaard[1], Barbara Ann Halkier[2] & Uffe Hasbro Mortensen[1]*

1) Eukaryotic Molecular Cell Biology, Section for Eukaryotic Biotechnology, Department of Systems Biology, Technical University of Denmark, Søltofts Plads, Building 223, 2800 Kongens Lyngby, Denmark
2) DynaMo Center, Department of Plant and Environmental Sciences, University of Copenhagen, Thorvaldsensvej 40, 1871 Frederiksberg C, Denmark

¥ - These authors contributed equally

* - Corresponding author, Email: um@bio.dtu.dk

Supplementary Information

Supplementary Figures

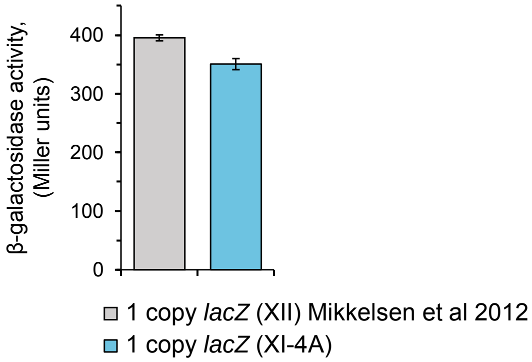

Supplementary Figure 1. Characterization of the new XI-4A integration site by measuring β-galactosidase activity. Error bars SD, n=3.

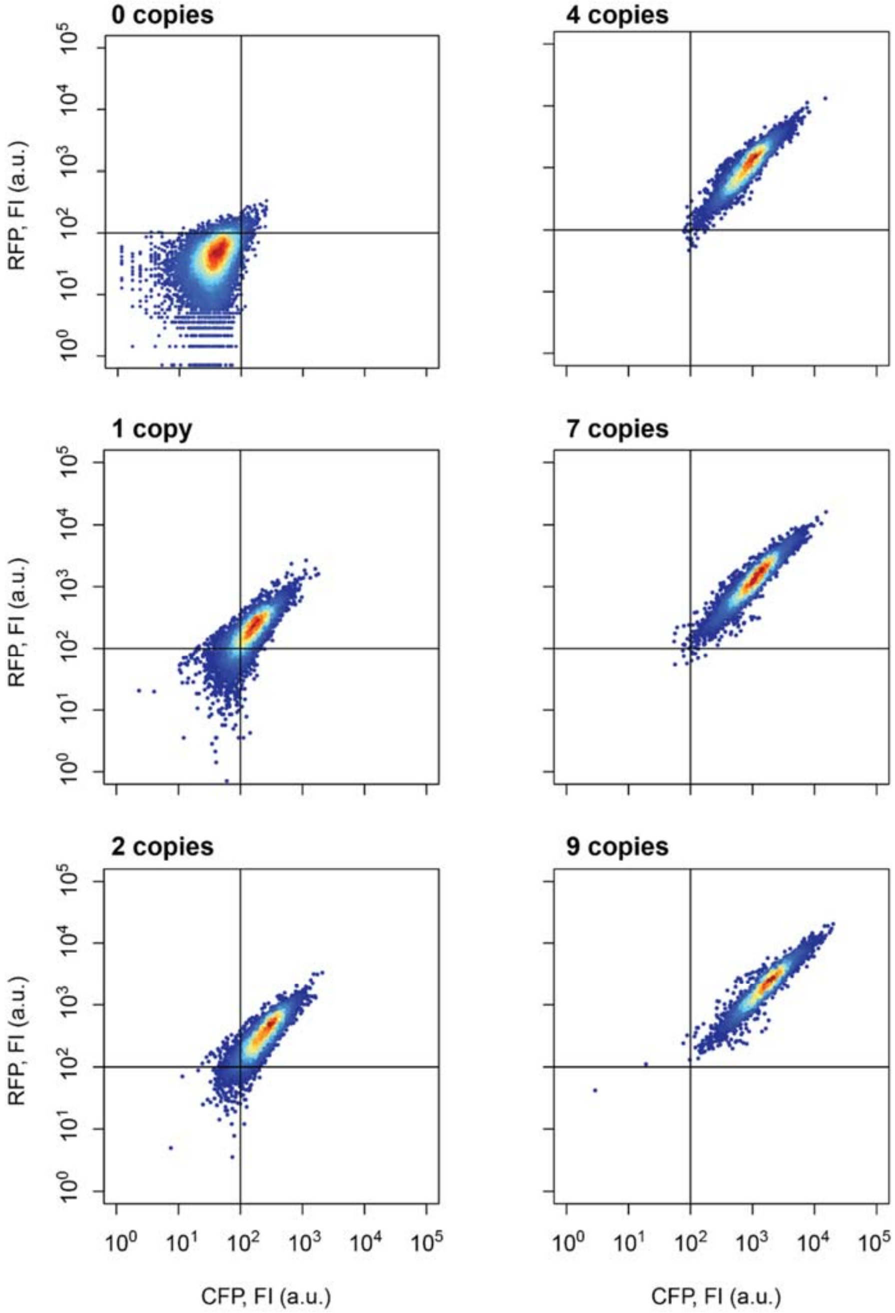

Supplementary Figure 2. Density scatter plots of the GA-X strains expressing various copies of the *CFP::RFP* gene pair.

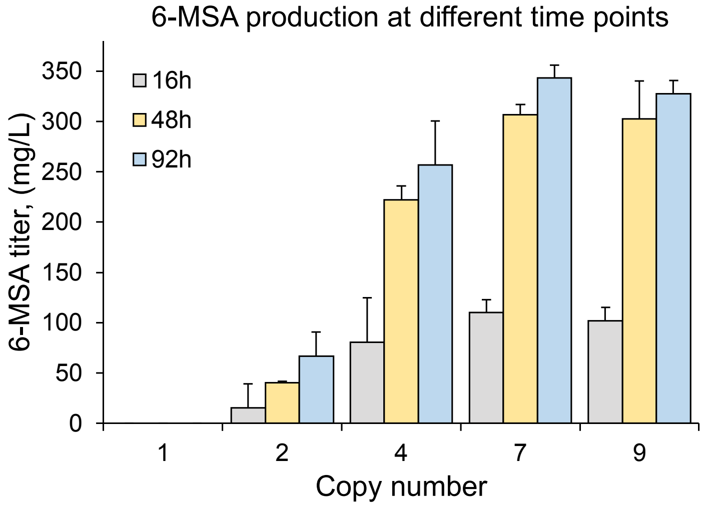

Supplementary Figure 3. 6-MSA titers at different time points of cultivation. Error bars represent SD, n=3.

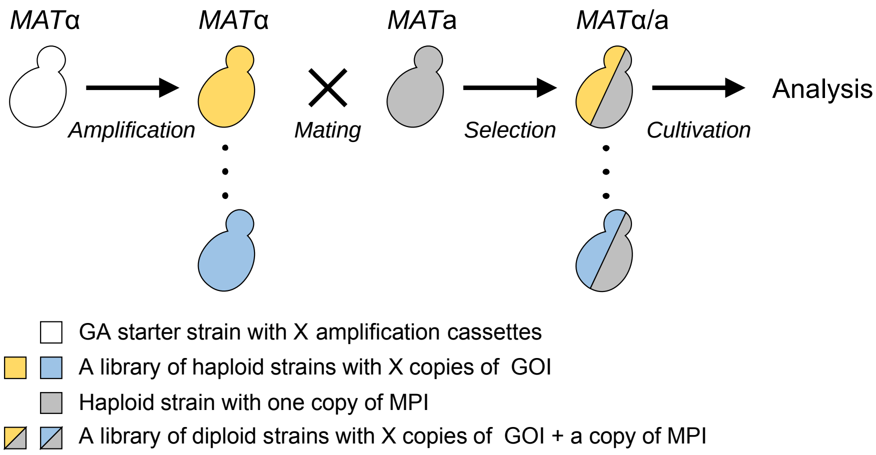

Supplementary Figure 4. Schematic representation of heterozygous diploid strain construction strategy used in this study. MPI – metabolic pathway of interest.

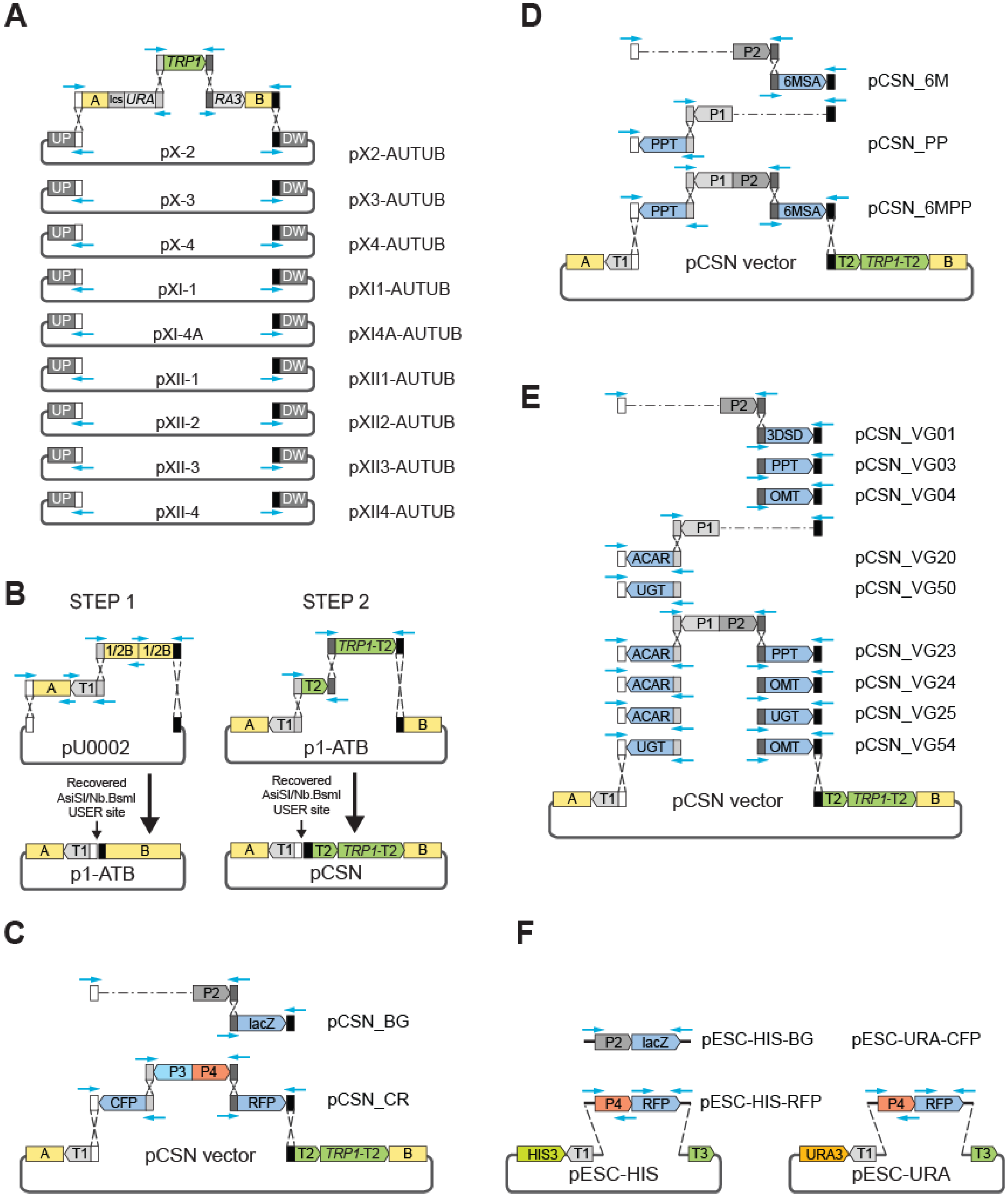

Supplementary Figure 5. Schematic overview of plasmid construction. **A**, assembly of the basic vectors for construction of the CASCADE starter strains (GAS-X). **B**, pCSN plasmid assembly steps. **C**, Construction of plasmids for proof-of-concept strains. **D**, Plasmids for 6-MSA experiments. **E**, plasmids for VG experiments. **F**, assembly of self-replicating multicopy plasmids. P1 – *pPGK1,* P2 – *pTEF1,* P3 – *pTPI1 and* P4 – *pACT1* promoters. T1 – *tADH1*, T2 – *tTRP1* and T3 – *tCYC1* terminators.

Supplementary Information

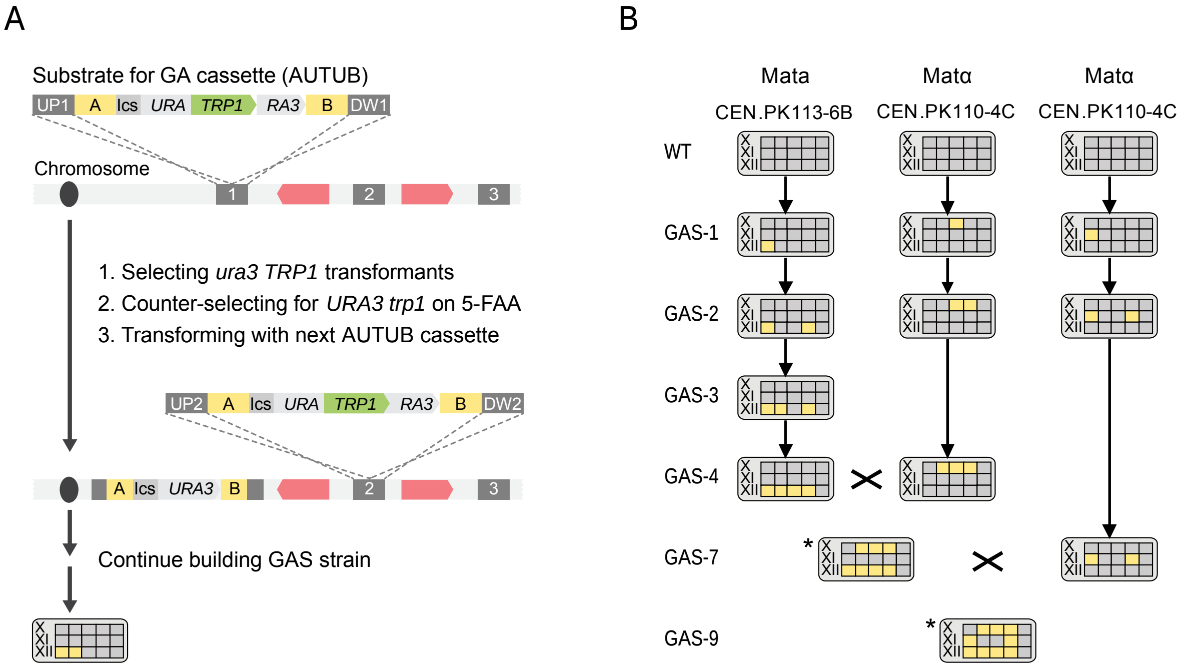

Supplementary Figure 6. Strategy for construction of the CASCADE starter strains GAS-X. **A**, A schematic representation of the sequential introduction of the GA cassettes into defined chromosomal locations by genetic transformation. **B**, combining clusters with GA cassettes by sexual crosses for construction of strains with up to 9 copies of GA.

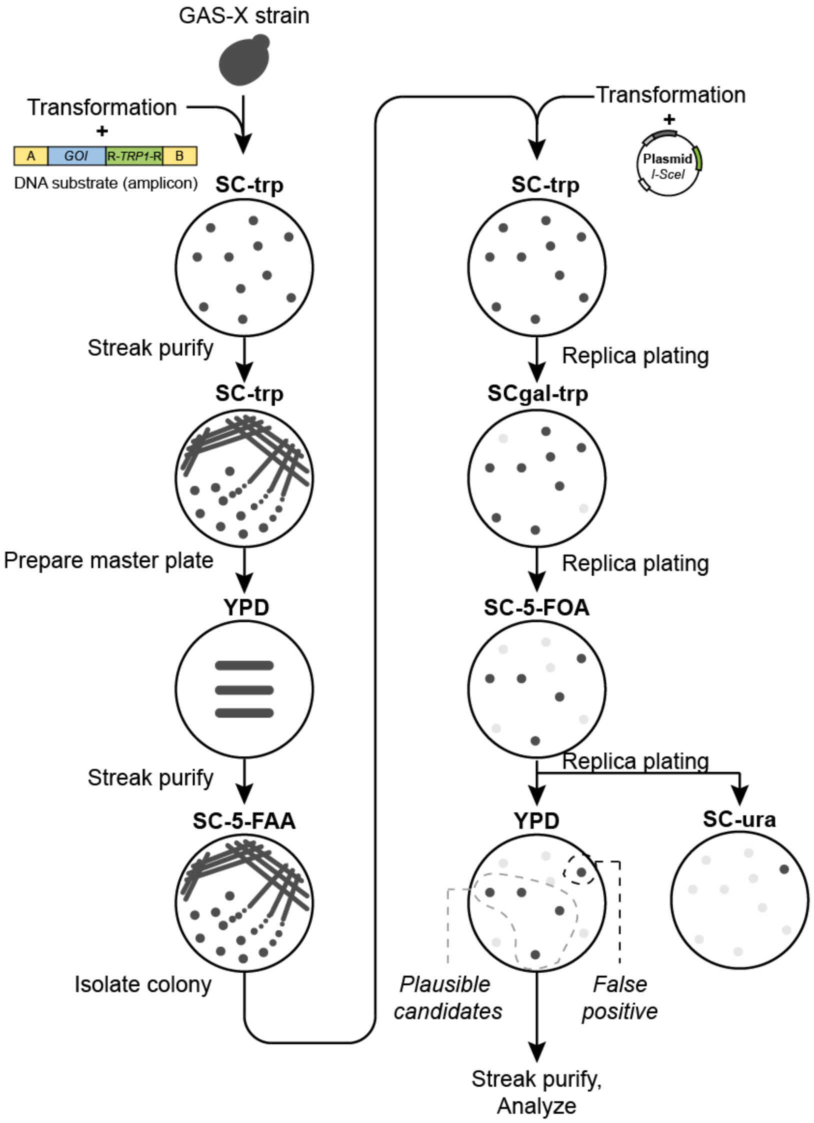

Supplementary Figure 7. Schematic protocol for gene amplification by CASCADE.

Supplementary Note: CFP and RFP production stability in long term cultivations

In this study we have investigated the stability of CFP and RFP production in a strain with nine integrated copies of the *CFP*::*RFP* gene pair (GA9-CR) and in a strain where the *CFP* and *RFP* genes are harbored on two different 2µ plasmids (2M-CR). The GA9-CR strains were cultivated in SC medium whereas the 2M-CR strains were cultivated in SC and in SC-his-ura medium for approx. 40 generations in shake flasks. Samples for flow cytometry were taken at regular intervals to assess the stability of fluorescent protein production.

Specifically, we measured both the mean fluorescence levels of CFP and RFP and the percentage of the cells that displayed fluorescence for both colors. The GA strain with nine copies of *CFP* and *RFP* genes retained its initial MFIs after 40 duplications in SC medium **Supplementary Figure 8Supplementary Figure 8**a. In contrast, with 2M-CR strains, MFIs were dropping right from the start of experiment in case of *CFP* (*URA3* plasmid) and after approx. 20 generations in case of *RFP* (*HIS3* plasmid). We note that with 2M-CR strains protein production was stable on selective SC-his-ura medium. Importantly, when co-expression of the *CFP* and *RFP* genes was evaluated (see **Supplementary Figure 8**b), we find that close to 100% of cells in the populations of GA9-CR strain contain CFP and RFP throughout the experiment in SC medium. For 2M-CR strains, only around 60 % of the cells contain both fluorescent proteins in selective medium; and this ratio appears constant at all time points of the experiment. In contrast, when 2M-CR is cultivated on non-selective medium, only 12% of the cells co-expressed both fluorescent proteins after 10 generations, and this number dropped to less 1% after 42 generations.

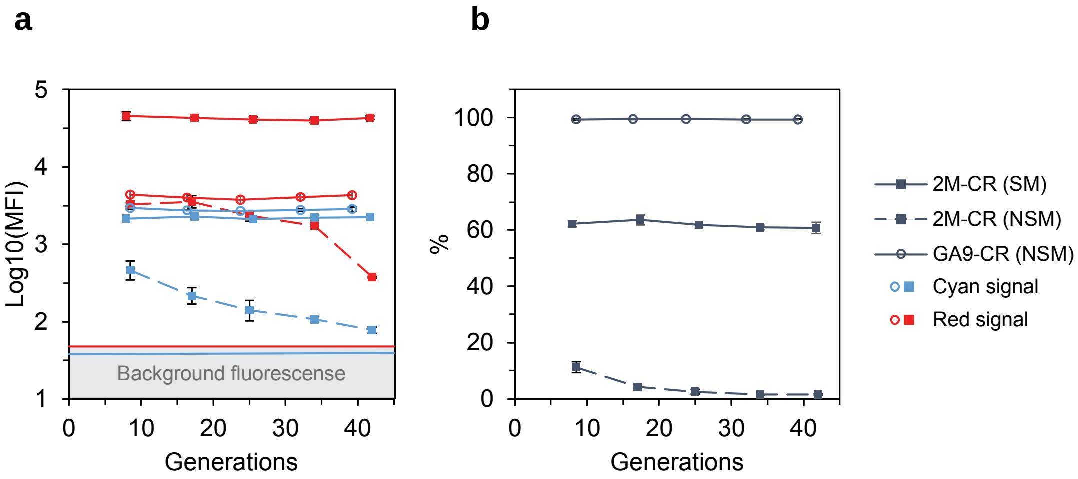

Supplementary Figure 8. Genetic stability of the GA strains compared to the multi-copy plasmid based expression systems. (**a**) Log_{10} MFI of the cells expressing *CFP* or *RFP* in two systems. The grey bar with red and blue lines at the bottom of the graph represent base line fluorescence levels for RFP and CFP signals, respectively. (**b**) Percentage of the cells that displayed fluorescence for both colors. SM and NSM represent Selective SC-his-ura medium and non-selective SC medium, respectively. Error bars show SD, n=3.

Supplementary Tables

Supplementary Table 1. Average percentage of the cell population expressing fluorescent proteins (n=3, biological replicates).

Copies	Average % of all population			
	None	CFP	RFP	CFP+RFP
Control	93.6	1.7	3.5	1.2
1	17.8	10.9	9.4	61.9
2	3.0	4.1	2.9	90.0
4	0.2	0.4	0.7	98.7
7	0.1	0.1	2.2	97.6
9	0.1	0.0	0.7	99.2
2μ	7.4	3.2	35.4	54.0

Supplementary Table 2. VG production profile values (expressed in percent) in the CASCADE strains as compared to reference strain. Standard deviation is based on 2 biological replicates.

Strain	PAC	PAL	VAC	VAN	VG	VAL	IVAC	IVAN	SUM
D1_VG_00	40.57 ± 1.94	1.59 ± 0.50	23.52 ± 2.35	0.00 ± 0.00	17.68 ± 4.91	5.28 ± 0.92	11.29 ± 3.33	0.06 ± 0.11	100.00
D8_VG_01	180.67 ± 0.05	5.37 ± 1.33	39.93 ± 0.28	0.00 ± 0.00	26.67 ± 11.46	5.36 ± 0.95	15.55 ± 2.01	0.58 ± 0.82	274.13
D8_VG_20	66.21 ± 8.78	18.00 ± 1.77	10.22 ± 3.58	2.35 ± 3.33	37.75 ± 3.37	8.90 ± 0.20	6.85 ± 0.47	3.58 ± 1.75	153.85
D8_VG_03	41.42 ± 9.12	2.28 ± 0.04	27.22 ± 16.70	0.00 ± 0.00	35.57 ± 8.62	10.41 ± 3.00	13.79 ± 6.08	0.46 ± 0.14	131.14
D8_VG_04	34.91 ± 2.97	2.47 ± 0.13	22.46 ± 2.81	0.00 ± 0.00	28.40 ± 2.54	10.79 ± 0.71	10.22 ± 0.09	0.68 ± 0.02	109.92
D8_VG_50	63.71 ± 3.19	0.66 ± 0.18	11.17 ± 0.82	0.00 ± 0.00	30.33 ± 3.49	0.94 ± 0.17	5.08 ± 7.18	0.00 ± 0.00	111.88
D8_VG_23	71.25 ± 31.94	16.72 ± 3.18	0.00 ± 0.00	4.01 ± 3.22	48.08 ± 29.10	7.50 ± 3.05	4.48 ± 0.05	2.60 ± 1.07	154.65
D8_VG_24	70.82 ± 9.39	13.57 ± 4.30	5.33 ± 0.15	15.48 ± 0.98	69.12 ± 18.54	18.55 ± 2.31	4.29 ± 0.51	4.54 ± 0.47	201.70
D8_VG_25	65.30 ± 11.55	14.77 ± 11.66	3.05 ± 0.46	4.07 ± 2.60	105.99 ± 12.36	4.78 ± 0.67	4.73 ± 0.53	1.55 ± 1.10	204.24
D8_VG_54	49.65 ± 5.56	1.99 ± 1.11	16.21 ± 2.32	1.97 ± 2.78	73.22 ± 32.92	6.67 ± 3.40	10.64 ± 1.46	0.91 ± 1.28	161.25

Supplementary Table 3. Sizes of DNA amplicons used in this study.

Plasmid	Amplicon size (w/o *TRP1*), bp	Total DNA amplified (nine copies), bp
pCSN-VG01	3,840	34,560
pCSN-VG20	6,813	61,317
pCSN-VG03	3,366	30,294
pCSN-VG04	3,403	30,627
pCSN-VG50	4,734	42,606
pCSN-VG23	7,891	71,019
pCSN-VG24	7,937	71,433
pCSN-VG25	8,717	78,453
pCSN-VG54	5,843	52,587
pCSN-BG	5,838	52,542
pCSN-CR	5,020	45,180
pCSN-6MPP	10,070	90,630
pCSN-6M	8,045	72,405
pCSN-PP	4,303	38,727

Supplementary Table 4. List of primers used in this study. All sequences are presented in 5' to 3' direction.

Name	Sequence
	**For pXI-4A construction**
P01	GGGTTTAAUGTGAATACTGCACAAAATGAATAC
P02	GGACTTAAUCTTGAATCTTTTAAGCAGCCA
P03	GGCATTAAUATCAAGAGAAACATTACAAGGC
P04	GGTCTTAAUCTGTGGCAAGATTGGCAAG
	**For AUTUB plasmid backbone amplification**
P05	ACGTAGCUACGTGACCACTTCGAGAGCA
P06	ACCTTGCUCCTGCATAATCGGCCTCACA
P07	ACGTAGCUAGTCTCGTATGTCGGCTCTC
P08	ACCTTGCUTGTGTCCGCGTTTCTAAGGC
P09	ACGTAGCUCTGCTCTTGAATGGCGACAG
P10	ACCTTGCUAACAGGCATGGGAAGATTCGC
P11	ACGTAGCUGGCTGCAAAGAATCCTCCGAG
P12	ACCTTGCUCTTCCACGGAATACCAAGCCC
P13	ACGTAGCUCTTGAATCTTTTAAGCAGCCA
P14	ACCTTGCUATCAAGAGAAACATTACAAGGC

P15	ACGTAGCUAATCAGACGCACGCTTGGCG
P16	ACCTTGCUTACGTGGATTGAGCCAGCAATAC
P17	ACGTAGCUGAAAGAACCGAACCGATGCCA
P18	ACCTTGCUCTTCCCGTGAATCAACTGCAC
P19	ACGTAGCUCATAACGCGTTACACGGAAGG
P20	ACCTTGCUCTACTATCGGCGACTCTCTC
P21	ACGTAGCUGTAATGCGAATGAGCAGGTAC
P22	ACCTTGCUGGAAGTTTTGCAGATGAAGTGC
P23	ACGTAGCUCTATCGCTGAACAGGAACTTAAG
P24	ACCTTGCUCCAACTTTGTACTATTCCTTCCC
P25	ACCTTGCUCTTCCCGTGAATCAACTGCAC
	**For AUTUB cassette assembly**
P26	AGCTACGUAGGTGTAAAAGTAGGGAGCG
P27	ACGAATGCUGAGCAATGAACCCAATAACGAAATC
P28	AGCATTCGUTGGCTTAACTATGCGGCATC
P29	AGTAGCATUCCTTTGACGTTGGAGTCCAC
P30	AATGCTACUCTTGACGTTCGTTCGACTGATGAGC
P31	AGCAAGGUGAGGAGAGTGGATGGATAGTC
	**For the bi-partite fragment assembly targeting the site VII**
P32	TAGCATACATGGCACCACTC
P33	CGCTCCCTACTTTTACACCTCACTATAAGGGTTCCTTCGGG
P34	GACTATCCATCCACTCTCCTCAACCGGAGTTGTCAGCAGAG
P35	ATGTATGGCAACGACAGCAG
P36	AGTTACGCTAGGGATAACAGGGTAATATAGCGCGTGATTCTGGGTAGAAGATC
P37	CAGGAGTAGCGTACATAGGATCATGGTGGTCAGCTGGAAT
P38	CTTGACGTTCGTTCGACTGATGAGC
P39	GAGCAATGAACCCAATAACGAAATC
P40	CTGAGGCTGAGGCGCCAGGTGTAAAAGTAGGGAGCG
P42	CGCTATATTACCCTGTTATCCCTAGCGTAACTTCACTGTCCGCTTGCAGAGT
P43	TCCTATGTACGCTACTCCTG
P44	GACGAAGAGCTCGAGGAGAGTGGATGGATAGTC
	**For pCSN construction**
P45	CGTGCGAUAGGTGTAAAAGTAGGGAGCG
P46	TCACTGTCCGCTTGCAGAGT
P47	ACTCTGCAAGCGGACAGTGAAGGGAAGAAAGCGAAAGGAG
P48	ATGCACGCGAUCGCACGCATTCGGGTCAAATCGTTGGTAGATACG
P49	ATCGCGTGCAUTCCTATCTTATGCCTTCATTTC
P50	CAGGAGTAGCGTACATAGGACAAAGCCCATTTCAATGAGC
P51	TCCTATGTACGCTACTCCTG
P52	CACGCGAUGAGGAGAGTGGATGGATAGTC
P53	AGTCAGTUTGGCTTAACTATGCGGCATC
P56	CACGCGAUCCTTTGACGTTGGAGTCCAC
P57	CGTGCGAUGAATGCGTGCGATCGCGTGCATTCGTTTGTGCACTTGCCTATGC
P58	AACTGACUCCTTTGACGTTGGAGTCCAC
	**For amplification of promoter regions**
P59	ACCCGTTGAUGCCGCTTGTTTTATATTTGTTG
P60	CGTGCGAUGCCGCACACACCATAGCTTC
P61	ACGTATCGCUGTGAGTCGTATTACGGATCCTTG
P62	ACTCATTUTTAGTTTATGTATGTGTTTTTTGTAG
P63	ACGAATGCUCTACGTATGGTCATTTCTTCTTCAG
P64	AGCATTCGUCAATATTTCTCTGTCACCCG

P65	ATGTTAAUTCAGTAAATTTTCGATCTTGG
	**For cloning of *lacZ, CFP* and *RFP* genes into pCSN**
P66	CGTGCGAUCACACACCATAGCTTCAAAATGTTTCTAC
P67	CACGCGAUTTATTTTTGACACCAGACCAACTGG
P68	CGTGCGAUTTATTTGTATAGTTCATCCATGCCATG
P68	AAATGAGUAAAGGAGAAGAACTTTTCACTG
P69	ATTAACAUGGCCTCCTCCGAGGACGT
P70	CACGCGAUCTAGGCGCCGGTGGAGTGGCGG
	**For cloning of 2micron based plasmids**
P71	TGATGAAGGATCCCACACACCATAGCTTCAAAATGTTTCTAC
P72	ATAACTCGGCCGCTTATTTTTGACACCAGACCAACTGG
P73	TATGGAAGGATCCCTACGTATGGTCATTTCTTCTTC
P74	CAGTGAAAAGTTCTTCTCCTTTACTCATTTTTAGTTTATGTATGTGTTTTTTGTAG
P75	ATGAGTAAAGGAGAAGAACTTTTCACTG
P76	ATAACTCGGCCGCTATTTGTATAGTTCATCCATGCCATG
P77	CGTTATTATACGGCCGATTTCTCTGTCACCCGGCCT
P78	AGGAGGCCATTGTTAATTCAGTAAATTTTCGATCTTGGGAAG
P79	TGAATTAACAATGGCCTCCTCCGAGGACGT
P80	TATTCAGATAGTCGACCTAGGCGCCGGTGGAGTGGCGG
	**For cloning of 6-MSA pathway genes into pCSN**
P81	ACAAATGCAUTCCGCTGCAACTTCTACA
P82	CACGCGAUTTAATGGTGATGGTGATGATGTT
P83	CGTGCGAUTTAGGATAGGCAATTACACACC
P84	ACAAATGGUGCAAGACACATCAAGCGC
	**For cloning of VG genes into pCSN**
P85	AGCGATACGUAAAAATGCCTTCCAAACTCGCC
P86	CACGCGAUTTACAAAGCCGCTGACAGC
P87	ATCAACGGGUAAAAATGGCTGTTGATTCACCAGATG
P88	CGTGCGAUCTTATAACAATTGTAACAATTCCAAATC
P89	AGCGATACGUAAAAATGGGTGACACTAAGGAGCAA
P90	CACGCGAUCTTATGGACCAGCTTCAGAACC
P91	AGCGATACGUAAAAATGGTCGATATGAAAACTACGC
P92	CACGCGAUTTAATCGTGTTGGCACAGC
P93	ATCAACGGGUAAAAATGCATATCACAAAACCACACG
P94	CGTGCGAUACTAGGCACCACGTGACAAGTC
	**Primers for marker swap in pWJ1320**
P95	TAGTGCAGCCTGCAGGGAGAGTGCACCATAGATCACG
P96	ACTCGATCTATCTCGAGTCCTTTGACGTTGGAGTCCAC
	**Verification primers for correct gene amplification**
P97(X2-F)	TGCGACAGAAGAAAGGGAAG
P98(X3-F)	TGACGAATCGTTAGGCACAG
P99(X4-F)	CTCACAAAGGGACGAATCCT
P100(XI1-F)	CTTAATGGGTAGTGCTTGACACG
P101(XI4A-F)	AAGCCCTATTATTGCTGACTTG
P102(XII1-F)	CTGGCAAGAGAACCACCAAT
P103(XII2-F)	CGAAGAAGGCCTCCAATTC
P104(XII3-F)	TGGGCAGCCTTGAGTAAATC
P105(XII4-F)	GAACTGACGTCGAAGGCTCT
P106(X2-R)	GAGAACGAGAGGACCCAACAT
P107(X3-R)	CCGTGCAATACCAAAATCG
P108(X4-R)	GACGGTACGTTGACCAGAG

P109(XI1-R)	GAAGACCCATGGTTCCAAGGA
P110(XI2-R)	GAGACAAGATGGGGCAAGAC
P111(XI3-R)	CACATTGAGCGAATGAAACG
P112(XII1-R)	GGACGACAACTACGGAGGAT
P113(XII2-R)	GGCCCTGATAAGGTTGTTG
P114(XII3-R)	TGGCCAATTGTTCAGTCAAG
P115(XII4-R)	CGTGAAATCTCTTTGCGGTAG
P116(generic)	CTTGAGTAACTCTTTCCTGTAGGTC

Supplementary Table 5. List of all plasmids constructed and used in this study.

Name	Description	Reference
	**Plasmids for GAS strain construction**	
pX-2	Backbone targeting to X2	[1]
pX-3	Backbone targeting to X3	[1]
pX-4	Backbone targeting to X4	[1]
pXI-1	Backbone targeting to XI1	[1]
pXI-4A	Backbone targeting to XI4A	This study
pXII-1	Backbone targeting to XII1	[1]
pXII-2	Backbone targeting to XII2	[1]
pXII-3	Backbone targeting to XII3	[1]
pXII-4	Backbone targeting to XII4	[1]
pWJ1042	*Template for TRP1*	[2]
pX2-AUTUB	X2::A-IsecI-URA-TRP1-RA3-B	This study
pX3-AUTUB	X3::A-IsecI-URA-TRP1-RA3-B	This study
pX4-AUTUB	X4::A-IsecI-URA-TRP1-RA3-B	This study
pXI1-AUTUB	XI1::A-IsecI-URA-TRP1-RA3-B	This study
pXI4A-AUTUB	XI4::A-IsecI-URA-TRP1-RA3-B	This study
pXII1-AUTUB	XII1::A-IsecI-URA-TRP1-RA3-B	This study
pXII2-AUTUB	XII2::A-IsecI-URA-TRP1-RA3-B	This study
pXII3-AUTUB	XII3::A-IsecI-URA-TRP1-RA3-B	This study
pXII4-AUTUB	XII4::A-IsecI-URA-TRP1-RA3-B	This study
	**Plasmids for pCSN construction**	
pU0002	AsiSI/Nb.BsmI cassette	[3]
pRS414	CEN/ARS, *TRP1*	[4]
p1-ATB	Intermediate for pCSN	This study
pCSN	A, AsiSI/Nb.BsmI, *R-TRP1-R*, B	This study
	**Proof of concept experiments**	
pSP-G2	Template for *pPGK1-pTEF1*	[5]
pWJ1042-AZC	Template for *lacZ*	[6]

Name	Description	Reference
pESC-HIS	2µ, *HIS3*	Agilent
pESC-URA	2µ, *URA3*	Agilent
pCSN-BG	A, *pTEF1::lacZ, R-TRP1-R*, B	This study
pESC-HIS-BG	2µ, *HIS3, pTEF1::lacZ*	This study
pWJ1163	Template for *CFP*	[2]
pWJ1350	Template for *RFP*	[7]
pCSN-CR	A, *pTPI1::CFP, pACT1::RFP, R-TRP1-R*, B	This study
pESC-HIS-RFP	2µ, *HIS3,* p*ACT1::RFP*	This study
pESC-URA-CFP	2µ, *URA3,* p*TPI1::CFP*	This study
	**6-MSA experiment**	
pRS426CTMSA-PP	Template for *6-MSAS* from *P. patulum*	[8]
pRS424CTnpgA	Template for *npgA* (PPTase) *from A. nidulans*	[8]
pXI2-6MPP	*XI2 site, pPGK1::npgA, pTEF1::6-MSAS, R-URA3-R*	This study
pCSN-6MPP	A, *pPGK1::npgA, pTEF1::6-MSAS, R-TRP1-R*, B	This study
pCSN-6M	A, *pTEF1::6-MSAS, R-TRP1-R*, B	This study
pCSN-PP	A, *pPGK1::npgA, R-TRP1-R*, B	This study
	**VG titration experiment**	
pJH500	Template for *3DSD* from *P.anserina*	[9]
pJH674	Template for *ACAR* from *Nocardia iowensis*	[9]
pJH589	Template for *EntD* (PPTase) *from E.coli*	[9]
pJH543	Template for *OMT* from *H.sapiens*	[9]
pJH665	Template for *UGT72E2* from *A.thaliana*	[9]
pCSN-VG01	A, *pTEF1::3DSD, R-TRP1-R*, B	This study
pCSN-VG20	A, *pPGK1::ACAR, R-TRP1-R*, B	This study
pCSN-VG03	A, *pTEF1::EntD, R-TRP1-R*, B	This study
pCSN-VG04	A, *pTEF1::OMT, R-TRP1-R*, B	This study
pCSN-VG50	A, *pPGK1::UGT72E2, R-TRP1-R*, B	This study
pCSN-VG23	A, *pTEF1::ACAR, pPGK1::EntD, R-TRP1-R*, B	This study
pCSN-VG24	A, *pTEF1::ACAR, pPGK1::OMT, R-TRP1-R*, B	This study
pCSN-VG25	A, *pTEF1::ACAR, pPGK1::UGT72E2, R-TRP1-R*, B	This study
	**Inductive plasmids**	
pWJ1320	*pGAL1:IsceI, URA3*	[7]
pWJ1320-TRP	*pGAL1:IsceI, TRP1*	This study

Supplementary Table 6. List of strains constructed and used in this work. All strains are based on CEN.PK. The genotype of all strains is MAL2-8C SUC2 + (definition in the table). The colored square in the grey 3X5 cell array represents the location of the GA cassette or integrated gene(s).

Name	*MATa*	*MATα*	*his3*	*leu2*	*trp1*	*ura3*	Copy No.	Genomic map	Description or Reference
Basic strains									
CEN.PK110-4C		•	•		•	•	—	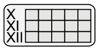	[10]
CEN.PK113-6B	•			•	•	•	—	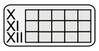	[10]
CEN.PK113-1C	•		•		•	•	—	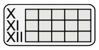	[10]
CEN.PK113-7A	•		•				—	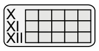	[10]
CEN.PK113-11C	•		•			•	—	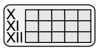	[10]
CEN.PK113-17A		•		•		•	—	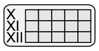	[10]
CEN.PK113-7B		•		•	•	•	—	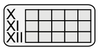	[10]
CEN.PK110-16D		•			•		—	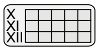	[10]
CEN.PK111-61A		•	•	•		•	—	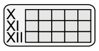	[10]
TS086		•			•	•	—	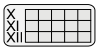	[11] (*Δadh6 Δbgl1)*
C-VG-aux	•		•			•	1	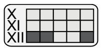	[11] Full VG pathway integrated on XII Chr.
GAS-X strains									
GAS-1A		•	•		•		1	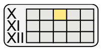	Constr. by transformation
GAS-1B		•	•		•		1	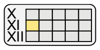	Constr. by transformation
GAS-1C	•			•	•		1	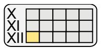	Constr. by transformation
GAS-1D	•		•		•		1	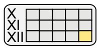	Constr. by transformation
GAS-2A		•	•		•		2	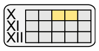	Constr. by transformation
GAS-2B	•			•	•		2	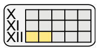	Constr. by transformation
GAS-2C	•			•	•		2	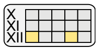	Constr. by transformation
GAS-2D	•		•		•		2	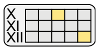	Constructed by cross
GAS-2E		•		•	•		2	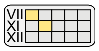	Contains VII site
GAS-2F		•	•		•		2	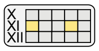	Constr. by transformation
GAS-3A		•	•		•		3	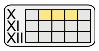	Constr. by transformation
GAS-3B	•			•	•		3	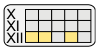	Constr. by transformation

Name	*MAT*a	*MAT*α	*his3*	*leu2*	*trp1*	*ura3*	Copy No.	Genomic map	Description or Reference
GAS-3C	•			•	•		3	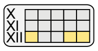	Constr. by transformation
GAS-4A	•			•	•		4	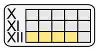	Constr. by transformation
GAS-4B,C		•		•	•		4	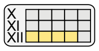	Constr. by transformation
GAS-4D	•		•		•		4	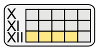	Constr. by transformation
GAS-4E		•			•		4	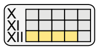	Constr. by transformation
GAS-7A, B, C		•		•	•		7	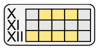	Constructed by cross
GAS-7D		•	•		•		7	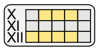	Constructed by cross
GAS-7E,F	•		•		•		7	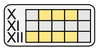	Constructed by cross
GAS-7G	•		•	•	•		7	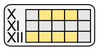	Constructed by cross
GAS-7VG		•			•		7	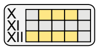	VG compatible (*adh6 bgl1*)
GAS-9A,B,C	•		•		•		9	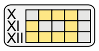	Constructed by cross
GAS-9D,E,F		•		•	•		9	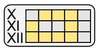	Constructed by cross
GAS-9G		•	•		•		9	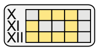	Constructed by cross
GAS-9H	•		•	•	•		9	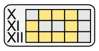	Constructed by cross
Proof of concept strains									
CEN.PK113-1C	•		•		•	•	0	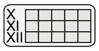	Negative control
GA1-BG	•		•		•		1	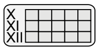	Not amplified GAS-4D with one copy of *lacZ* integrated
GA2-BG	•		•		•	•	2	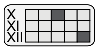	*lacZ* amplification
GA4-BG	•		•		•	•	4	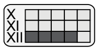	*lacZ* amplification
GA7-BG		•		•	•	•	7	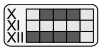	*lacZ* amplification
2M-BG	•				•	•	?	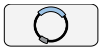	Multi copy plasmid expression (pESC-HIS-BG)
GA1-CR	•		•		•		1	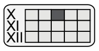	Not amplified GAS-7F with one copy of *CFP::RFP* integrated
GA2-CR	•		•		•	•	2	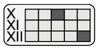	*CFP::RFP* amplification
GA4-CR	•		•		•	•	4	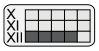	*CFP::RFP* amplification
GA7-CR	•		•		•	•	7	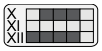	*CFP::RFP* amplification
GA9-CR	•		•		•	•	9	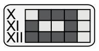	*CFP::RFP* amplification

Name	*MAT***a**	*MATα*	*his3*	*leu2*	*trp1*	*ura3*	Copy No.	Genomic map	Description or Reference
2M-CR	•				•		?	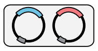	Multi copy plasmid expression (pESC-HIS-RFP, pESC-URA-CFP)
6-MSA strains									
XI2_6MPP		•		•		•	1	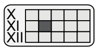	Basic 6-MSA producing strain
GA1-6MPP	•		•		•	•	1	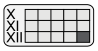	6-MSA pathway amplification
GA2-6MPP	•		•		•	•	2	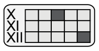	6-MSA pathway amplification
GA4-6MPP	•		•		•	•	4	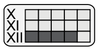	6-MSA pathway amplification
GA7-6MPP	•		•		•	•	7	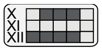	6-MSA pathway amplification
GA9-6MPP	•		•		•	•	9	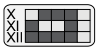	6-MSA pathway amplification
GA1-6M	•		•		•	•	1	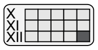	*6-MSAS* amplification
GA2-6M	•		•		•	•	2	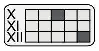	*6-MSAS* amplification
GA7-6M	•		•		•	•	7	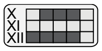	*6-MSAS* amplification
GA1-PP	•		•		•	•	1	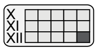	*npgA (PPT*) amplification
GA2-PP	•		•		•	•	2	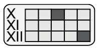	*npgA (PPT*) amplification
GA7-PP	•		•		•	•	7	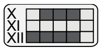	*npgA (PPT*) amplification
VG strains									
XII_VG	•		•				1	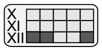	VG pathway
GA7-VG01		•			•	•	7	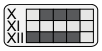	*3DSD* amplification
GA7-VG20		•			•	•	7	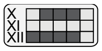	*ACAR* amplification
GA7-VG03		•			•	•	7	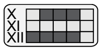	*EntD (PPT)* amplification
GA7-VG04		•			•	•	7	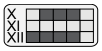	*OMT* amplification
GA7-VG50		•			•	•	7	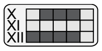	*UGT* amplification
GA7-VG23		•			•	•	7	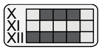	*ACAR+PPT* amplification
GA7-VG24		•			•	•	7	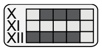	*ACAR+OMT* amplification
GA7-VG25		•			•	•	7	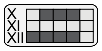	*ACAR+UGT* amplification
GA7-VG54		•			•	•	7	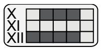	*UGT+OMT* amplification

Supplementary Table 7. The list of diploid strains constructed in this study.

**Name**	*MAT*<u>**a**</u> **strain**	*MAT*α **strain**	**Copies**	**Comments**
6-MSA titration				
D0-6-MSA	CEN.PK113-11C	CEN.PK113-17A	0+0	Negative control
D2-6-MSA	GA1-6M	XI2_6MPP	1+1	6-MSAS titration
D3-6-MSA	GA2-6M	XI2_6MPP	2+1	6-MSAS titration
D8-6-MSA	GA7-6M	XI2_6MPP	7+1	6-MSAS titration
D2-PPT	GA1-PP	XI2_6MPP	1+1	PPTase titration
D3-PPT	GA2-PP	XI2_6MPP	2+1	PPTase titration
D8-PPT	GA7-PP	XI2_6MPP	7+1	PPTase titration
VG titration				
D1-VG-00	XII_VG	TS086	1+0	Reference strain
D8-VG-01	XII_VG	GA7-VG01	1+7	*3DSD* amplification
D8-VG-20	XII_VG	GA7-VG20	1+7	*ACAR* amplification
D8-VG-03	XII_VG	GA7-VG03	1+7	*EntD (PPT*) amplification
D8-VG-04	XII_VG	GA7-VG04	1+7	*OMT* amplification
D8-VG-50	XII_VG	GA7-VG50	1+7	*UGT* amplification
D8-VG-23	XII_VG	GA7-VG23	1+7	*ACAR+PPT* amplification
D8-VG-24	XII_VG	GA7-VG24	1+7	*ACAR+OMT* amplification
D8-VG-25	XII_VG	GA7-VG25	1+7	*ACAR+UGT* amplification
D8-VG-54	XII_VG	GA7-VG54	1+7	*UGT+OMT* amplification

References

1. Mikkelsen, M. D. *et al.* Microbial production of indolylglucosinolate through engineering of a multi-gene pathway in a versatile yeast expression platform. *Metab. Eng.* **14,** 104–11 (2012).

2. Reid, R. J. D. R., Lisby, M. & Rothstein, R. Cloning-free genome alterations in saccharomyces cereuisiae using adaptamer-mediated PCR. *Methods Enzymol.* **350,** 258–277 (2002).

3. Hansen, B. G. *et al.* Versatile enzyme expression and characterization system for Aspergillus nidulans, with the Penicillium brevicompactum polyketide synthase gene from the mycophenolic acid gene cluster as a test case. *Appl. Environ. Microbiol.* **77,** 3044–51 (2011).

4. Sikorski, R. S. & Hieter, P. A System of Shuttle Vectors and Yeast Host Strains Designed for Efficient Manipulation of DNA in. *Genetics* 19–27 (1989).

5. Partow, S., Siewers, V., Bjørn, S., Nielsen, J. & Maury, J. Characterization of different promoters for designing a new expression vector in Saccharomyces cerevisiae. *Yeast* **27,** 955–964 (2010).

6. Flagfeldt, D. B., Siewers, V., Huang, L. & Nielsen, J. Characterization of chromosomal integration sites for heterologous gene expression in Saccharomyces cerevisiae. *Yeast* 545–551 (2009). doi:10.1002/yea

7. Lisby, M., Mortensen, U. H. & Rothstein, R. Colocalization of multiple DNA double-strand breaks at a single Rad52 repair centre. *Nat. Cell Biol.* **5,** 572–7 (2003).

8. Wattanachaisaereekul, S., Lantz, A. E., Nielsen, M. L. & Nielsen, J. Production of the polyketide 6-MSA in yeast engineered for increased malonyl-CoA supply. *Metab. Eng.* **10,** 246–254 (2008).

9. Hansen, E. E. H. *et al.* De novo biosynthesis of vanillin in fission yeast (Schizosaccharomyces pombe) and baker's yeast (Saccharomyces cerevisiae). *Appl. Environ. Microbiol.* **75,** 2765–74 (2009).

10. Entian, K. & Kötter, P. Yeast genetic strain and plasmid collections. *Methods Microbiol.* **36,** 629–666

(2007).

11. Strucko, T., Magdenoska, O. & Mortensen, U. H. Benchmarking two commonly used Saccharomyces cerevisiae strains for heterologous vanillin-β-glucoside production. *Metab. Eng. Commun.* **2,** 99–108 (2015).
